# Enabling precision medicine by unravelling disease pathophysiology: quantifying signal transduction pathway activity across cell and tissue types

**DOI:** 10.1038/s41598-018-38179-x

**Published:** 2019-02-07

**Authors:** Anja van de Stolpe, Laurent Holtzer, Henk van Ooijen, Marcia Alves de Inda, Wim Verhaegh

**Affiliations:** Philips Research, High Tech Campus 11, 5656 AE Eindhoven, The Netherlands

## Abstract

Signal transduction pathways are important in physiology and pathophysiology. Targeted drugs aim at modifying pathogenic pathway activity, e.g., in cancer. Optimal treatment choice requires assays to measure pathway activity in individual patient tissue or cell samples. We developed a method enabling quantitative measurement of functional pathway activity based on Bayesian computational model inference of pathway activity from measurements of *mRNA levels of target genes of the pathway-associated transcription factor*. Oestrogen receptor, Wnt, and PI3K-FOXO pathway assays have been described previously. Here, we report model development for androgen receptor, Hedgehog, TGFβ, and NFκB pathway assays, biological validation on multiple cell types, and analysis of data from published clinical studies (multiple sclerosis, amyotrophic lateral sclerosis, contact dermatitis, Ewing sarcoma, lymphoma, medulloblastoma, ependymoma, skin and prostate cancer). Multiple pathway analysis of clinical prostate cancer (PCa) studies showed increased AR activity in hyperplasia and primary PCa but variable AR activity in castrate resistant (CR) PCa, loss of TGFβ activity in PCa, increased Wnt activity in TMPRSS2:ERG fusion protein-positive PCa, active PI3K pathway in advanced PCa, and active PI3K and NFκB as potential hormonal resistance pathways. Potential value for future clinical practice includes disease subtyping and prediction and targeted therapy response prediction and monitoring.

## Introduction

Signal transduction pathways control basic cellular processes such as cell division, differentiation and migration and play important roles in disease pathophysiology^1–4^. They can be categorized as hormonal nuclear receptor pathways (e.g., androgen and oestrogen receptor pathways), developmental pathways (e.g., Wnt, Hedgehog, and TGFβ pathways), growth factor pathways (e.g., PI3K pathway) and immune pathways (e.g., NFκB pathway).

During the past few decades, targeted drugs have become clinically available. They aim at correcting disease pathophysiology and ‘target’ specific locations in signalling pathways to modify pathway activity. While frequently developed for the treatment of cancer and immune-mediated diseases, treatment of other diseases is being pioneered. Depending on the clinical application, the goal of treatment can be to increase specific pathway activity (for example to activate specific immune cells) or to inhibit it (in case of a tumour-driving signalling pathway in a cancer). In general, only a subset of patients with a specific disease responds to a targeted drug, and choosing the right drug for a patient is a major challenge. Developing diagnostic assays to reliably predict therapy response has proven difficult. Efforts to predict response in cancer patients based on genome mutation analysis fail in the majority of patients despite being effective in selected cases^5^.

Assays that measure *functional activity of signal transduction pathways* in a cell/tissue sample are expected to improve prediction of therapy response. During the past decade, we have developed a new computational approach to quantify signal transduction pathway activity in individual cell or tissue samples based on measurements of *mRNA levels of direct target genes of a transcription factor* belonging to a respective signalling pathway^6,7^. The general concept and development of the first pathway computational models (Wnt, ER, and PI3K pathways) have been described previously^6,8^. We describe here the development of similar computational pathway models for quantitative measurement of activity of the androgen receptor (AR), Hedgehog (HH), TGFβ, and NFκB pathways.

The androgen receptor is a member of the nuclear receptor family, and upon binding androgens such as testosterone or dihydrotestosterone (DHT), it becomes transcriptionally active^9,10^. Physiological activation of the HH pathway results from ligand (e.g., Sonic Hedgehog, SHH) binding to the PTCH membrane receptor, leading to activation of the GLI transcription factor and transcription of GLI target genes^11–13^. TGFβ ligands bind membrane TGFβ-type II receptors to recruit TGFβ-type I receptors and induce a transcription factor complex composed of an R-SMAD (SMAD2, SMAD3) and SMAD4, initiating transcription of target genes^14–16^. The transcription factor of the nuclear factor kappa-light-chain-enhancer of activated B cells (NFκB) pathway is typically activated by a cytokine such as TNFα^17,18^. All these signalling pathways can be activated in disease by abnormal availability of ligand, abnormal crosstalk with another pathway, e.g., the PI3K pathway, or  mutations in key pathway genes^3,16,19^.

In addition to biological validation of the pathway model-based assays on a variety of cell and tissue types, example clinical studies have been analysed to illustrate potential clinical utility. First envisioned applications are cancer subtyping and therapy response prediction. We expect our models to also have potential for diagnosis, subtyping and management of other diseases as well as for drug development and life sciences applications.

## Methods

### Development of the Bayesian network models for the respective signal transduction pathways

**Figure 1:**
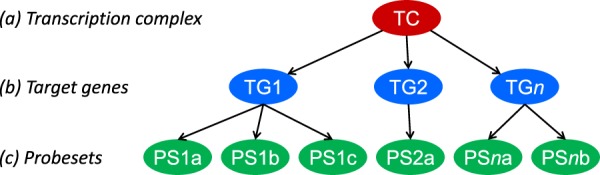
The structure of the Bayesian networks used to model the transcriptional programme of signalling pathways (with permission from^6^). The transcription complex (TC) refers to the transcription factor associated with a specific signal transduction pathway, which can be present in an inactive or active gene-transcribing state; target genes (TG) refer to direct target genes of the transcription complex; probesets (PS) refer to probesets for the respective target gene present on the Affymetrix HG-U133 Plus 2.0 microarray.

The mathematical approach to develop Bayesian network models for the measurement of signal transduction pathway activity has been described previously^6–8^. In brief, the computational network model for a pathway is constructed to infer the probability that the pathway-associated transcription factor is actively transcribing its target genes (Fig. 1). The Bayesian network describes (i) the causal relation that a target gene is up- or downregulated depending on the transcription complex being active or inactive and (ii) the causal relation that a probeset is high or low depending on the target gene being up or down. These relations are probabilistic in nature; the parameters describing relation (i) have been based on literature insights, and the parameters describing relation (ii) are based on calibration data of samples with ground truth information about their pathway activity state, as discussed below. More details can be found in an earlier publication^6^.

Target genes for AR, HH, TGFβ and NFκB pathway models were selected according to the same principles as described for Wnt and ER pathway models using available scientific literature. For each putative target gene, evidence was assessed for its gene promotor region containing a response element motif for the respective transcription factor, functionality of the respective promoter (e.g., in promoter-luciferase experiments), binding of the transcription factor to the respective response/enhancer element *in vivo* (e.g., ChIPseq) or *in vitro* (electrophoretic mobility shift assay, EMSA), and differential expression upon pathway activation. Based on this accumulated experimental evidence information, a ‘direct target gene evidence score’ was constructed, and candidate genes were ranked according to this score. For the final selection, consistency of evidence obtained by multiple expert research groups and on multiple cell types was taken into account (for details on target gene selection, see Supplemental information). Approximately 25–35 target genes per pathway were selected for creation of the computational pathway models. This number is high enough to ensure robustness and sensitivity of the pathway assay while limiting the selection to only the highest evidence target genes to enable maximal specificity. Importantly, target genes were only selected based on evidence for reproducible and specific transcription factor-induced transactivation across various cell types, irrespective of the function of encoded proteins, since we interpret target gene levels solely as evidence of transcription factor activity associated with a signalling pathway.

Probesets on the Affymetrix HG-U133Plus2.0 microarray associated with the target genes were selected based on the Bioconductor package available in R and manual curation using the latest information available on the UCSC Genome Browser (www.genome.ucsc.edu)^20^. Typical errors encountered during manual curation were alignment of probeset sequences with introns of the target genes and probesets positioned on the opposite strand, and in some rare cases, probesets were even found on completely different chromosomes than the respective target gene. During the same process, probesets that were missing in Bioconductor were added to the list. The final selection of probesets can be found in the Supplementary Information.

### Calibration of the Bayesian computational models

Since mRNA levels used as input for the models are absolute measurements, the Bayesian models need to be calibrated on cell or tissue samples in which the respective signal transduction pathway is known to be either active or inactive (the ‘ground truth’). For this purpose, Affymetrix data can be used from a variety of sources, such as cell line experiments, patient-derived xenograft (PDX) mice, or clinical samples, including data from public datasets (e.g., the Gene Expression Omnibus (GEO) database of preclinical or clinical studies). In the current study, models were only calibrated once and subsequently frozen and validated on a variety of independent datasets from various cell and tissue types of preclinical and clinical studies (list of datasets used in Supplemental Information).

After model parameters have been calibrated, they are frozen, and mRNA probeset measurements of a newly measured sample can be entered into the model, after which Bayesian inference is used to estimate the probability *P* that the respective pathway is active in the sample. Such a probability *P* may be used directly as read-out or first be transformed into a log2odds value log_2_(*P*/(1 − *P*)), as the latter may show more detail if *P* is close to either 0 or 1. The log2odds value is referred to as ‘Pathway activity score’, can be used in a quantitative manner to identify differences in pathway activity between samples, and is used in all figures and results in this paper. Optionally, for standardization purposes, the log2odds values can be converted to a 0–100 scale.

If pathway activity results are required in a discrete manner, i.e., as a yes-or-no answer, a threshold can either be set on the probability *P* or on the log2odds values (Supplemental information Fig. 1). A default threshold is set at a probability of 0.5, corresponding to a log2odds of 0. For test samples of a similar tissue type as the calibration samples, this has proven to be a valid approach. In general, for most signalling pathways, differences in absolute mRNA levels of target genes are not as large as expected between different tissue/cell types, enabling use of the models on a large variety of cell/tissue types without any model adaptation. Resetting of a threshold may be needed in case of use on another cell/tissue type or with a specific clinical question, but the model does not need to be adapted. Alternatively, if sufficient ‘ground truth’ data with respect to an active and inactive pathway are available on the cell or tissue type of interest, the model can be re-calibrated on the available sample set, for example, for specific use on the cell/tissue of interest. However, simply redefining the threshold has the advantage of keeping exactly the same model, enabling direct quantitative comparison between results obtained on different datasets, for example, obtained on completely different cell or tissue types.

### Validation of the Bayesian computational models and exploration of potential clinical utility

Following a single calibration step on a specific cell or tissue type, models were frozen, and each of the pathway models was biologically validated on independent Affymetrix HG-U133Plus2.0 microarray data from multiple independent cell line experimental and/or patient datasets, including data from other cell and tissue types than present in the calibration set. For each independent dataset used for validation purposes, the ‘ground truth’ with respect to signalling pathway activity was known. Subsequently, example clinical studies with Affymetrix datasets were analysed to illustrate potential clinical applications, and finally, a number of datasets from clinical studies on prostate cancer were used to illustrate the value of combined signal transduction pathway analysis, all on individual patient samples.

### Use of pathway models to measure activity of the Wnt, oestrogen receptor (ER) and PI3K pathways

On a number of prostate cancer datasets, a multiple pathway analysis was performed. In addition to the pathway models described here, the previously described Bayesian model for measurement of growth factor PI3K pathway activity, as well as the Bayesian models for oestrogen receptor (ER) and (canonical) Wnt pathways, were used as described previously^6,8^. The canonical Wnt pathway is activated by a Wnt ligand that binds to the Frizzled membrane receptor, ultimately resulting in nuclear translocation of the co-activator beta-catenin, which activates transcription through a TCF/LEF transcription factor^21^. The ER pathway is a nuclear receptor pathway and is activated by oestrogens that bind to and induce dimerization of intracellular ER proteins (ERα or ERβ) resulting in activation of the ER transcription factor^22^. The PI3K pathway is a growth factor pathway activated by growth factors such as Epidermal Growth Factor (EGF), resulting in activation of PI3K and Akt with consequent removal of the FOXO3 transcription factor from the nucleus and loss of its transcriptional activity^23,24^. Interpretation of analysis results of the Wnt and ER pathway models is straightforward since they measure transcriptional activity of β-catenin/TCF and ER transcription factor, respectively; for the PI3K pathway, this is more complex. In brief, the PI3K pathway model measures activity of the FOXO transcription factor, which is inversely related to PI3K pathway activity in the absence of cellular oxidative stress^25^. In the presence of oxidative stress, the FOXO transcription factor becomes alternatively phosphorylated and activated to include transcription of target genes that protect against oxidative stress, i.e., the Superoxide Dismutase 2 (SOD2) gene. The expression level of this FOXO target gene is measured in every sample based on Affymetrix probeset hybridization values and is used to distinguish between the two functional activity states of FOXO. For several tissue types, including prostate tissue, mean SOD2 levels in normal (non-oxidative stress) tissue have been determined, and an upper threshold for non-oxidative stress SOD2 was set at 2 SDs over the mean value^8^. In samples with active FOXO and elevated SOD2 expression level (>threshold), FOXO activity is considered as oxidative stress-induced. When FOXO is active in the oxidative stress mode, it does not function any more as a readout of PI3K pathway activity, and formally, no conclusion can be drawn on activity of this pathway^8^. In the analysed datasets, SOD2 levels were below the pre-determined oxidative stress threshold for prostate tissue and PI3K pathway activity could be directly inferred from the FOXO activity score^8^.

### Microarray data source and quality control

All calibration and validation datasets, as well as datasets used for exploration of potential clinical utility, consisted of Affymetrix HG-U133Plus2.0 data available from GEO (www.ncbi.nlm.nih.gov/geo). A list with all GEO datasets that were used, with associated publications, is available in the Supplemental Information.

Before using microarray data, extensive quality control (QC) was performed on Affymetrix data from each individual sample based on 12 different quality parameters following Affymetrix recommendations and previously published literature^26,27^. In summary, these parameters include the average value of all probe intensities, negative or extremely high (>16-bit) intensity values, poly-A RNA (sample preparation spike-ins) and labelled cRNA (hybridization spike ins) controls, *GAPDH* and *ACTB* 3′/5′ ratio, centre of intensity and values of positive and negative border controls determined by affyQCReport package in R, and an RNA degradation value determined by the AffyRNAdeg function from the Affymetrix package in R^28,29^. Samples that failed the QC process were removed from the analysis (Supplemental information, Table 1).

### Statistics

In principle, two-sided Wilcoxon signed-rank statistical tests were performed. In case another statistical method was more appropriate due to the content of a specific dataset, this is indicated in the legend of the figure. For pathway correlation statistics, both Pearson correlation and Spearman rank correlation tests were performed; since the results were similar, only the Pearson correlation coefficient and associated p value is reported.

## Results

### Development and validation of Bayesian models

#### HH pathway

**Figure 2:**
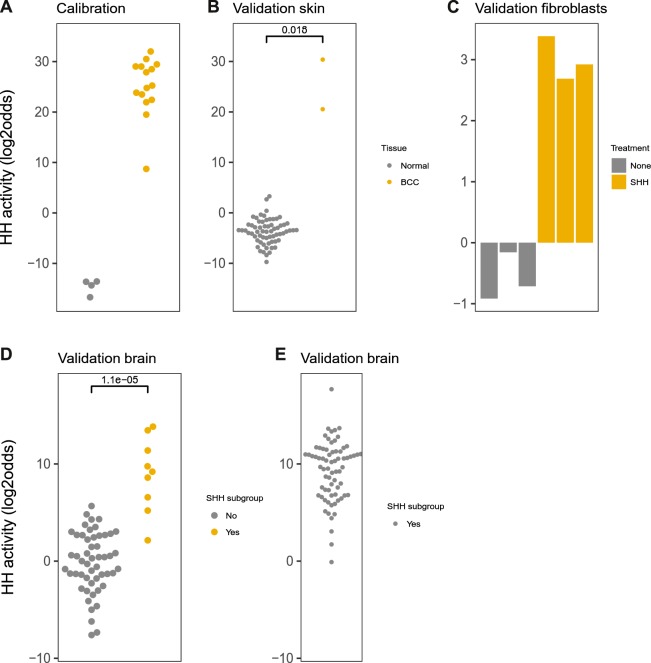
HH pathway model. (**A**) Calibration of the HH pathway model on dataset GSE7553 containing Affymetrix HG-U133Plus2.0 microarray data from tissue samples of basal cell carcinoma (BCC) and healthy skin (normal). (**B**–**F**) Validation on independent GEO datasets, different cell/tissue types. (**B**) GSE39612, tissue samples of basal cell carcinoma (BCC) and healthy skin. (**C**) GSE29316, Sonic Hedgehog-stimulated (SHH, 1 µg/ml) primary human colon myofibroblasts (CCD-18Co) in co-culture with HUVECs. (**D**) GSE37418, medulloblastomas with a separate Sonic Hedgehog (SHH) medulloblastoma subtype group. (**E**) GSE49243, SHH medulloblastoma subtype. The activity score is calculated as log2odds. Two-sided Wilcoxon signed-rank statistical tests were performed; p-values are indicated in the figures. In case fewer than 4 samples needed presentation, bar plots are used instead of dot blots.

Thirty-three high evidence direct GLI target genes were used to build the Bayesian model (Supplemental Information). The HH pathway is known to be active in basal cell carcinoma (BCC) and inactive in normal skin samples; hence, fifteen BCC and four healthy skin samples were used as active and inactive calibration samples, respectively. The resulting pathway scores on calibration data showed excellent separation between normal skin and BCC samples^30^ (Fig. 2A).

Subsequently, the pathway model was frozen and biologically validated on independent Affymetrix datasets from samples with known Hedgehog pathway activity status (Fig. 2B–E). Two BCC samples were available that showed the expected high activity, while additional normal skin samples had low HH activity (Fig. 2B). The Hedgehog pathway can be activated by the ligand Sonic Hedgehog (SHH) in fibroblasts^31^. Untreated fibroblasts had low HH pathway activity, which increased after stimulation with SHH ligand (Fig. 2C). In medulloblastoma, a presumably HH-active SHH subtype has been defined by a working group^32^. The model successfully separated SHH subtype from other subtypes, enabling definition of an activity threshold (log2odds = 5) for HH activity in brain tissue (Fig. 2D). Another clinical study contained only SHH subtype samples, of which 89% carried HH activating gene mutations^33^. Using the identified threshold, 93% (n = 76) fell into the HH-active category (Fig. 2E). In conclusion, even though calibrated on skin samples, the model also scored HH pathway activity correctly in other cell types for which ‘ground truth’ datasets were available. The results illustrate that a threshold for pathway activity can be defined without model adaptation.

#### TGFβ pathway

**Figure 3:**
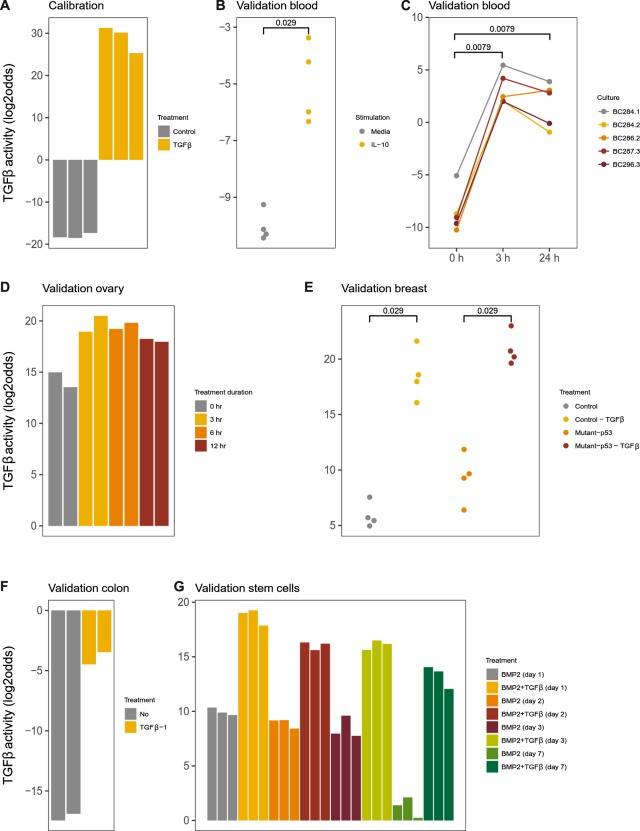
TGFβ pathway model. (**A**) Calibration of the TGFβ pathway model on dataset GSE17708 containing A549 lung adenocarcinoma cell line samples stimulated with 5 ng/mL TGFβ1. (**B–G**) Validation on independent GEO datasets, different cell types. (**B**) GSE43700, interleukin 10 (IL10) stimulation of peripheral blood mononuclear cells. (**C**) GSE7568, TGFβ1 (10 ng/ml) stimulation during indicated time periods of primary macrophages differentiated by treatment with dexamethasone; samples from 5 independent donors. (**D**) GSE6653, immortalized ovarian surface epithelial cells (IOSE) derived from normal ovarian epithelial cells stimulated with TGFβ1 (10 ng/ml) during time periods as indicated. (**E**) GSE14491, treatment with TGFβ1 (5 ng/ml) of breast cancer cell line MDA-MB-231, transfected with either control (shGFP) or anti-p53 (shp53) short-hairpin RNAs. (**F**) GSE59771, TGFβ1 treatment of a colon cancer cell line with inducible TGFβ1 R2 (no associated publication). (**G**) GSE84500, BMP2 treatment of human mesenchymal stem cells for 1, 2, 3 or 7 days in the presence or absence of TGFβ1 (2 ng/ml). The activity score is calculated as log2odds. Two-sided Wilcoxon signed-rank statistical tests were performed; p-values are indicated in the figures.

Twenty-eight target genes were selected for the model (Supplemental Information). The lung cancer cell line A549 is known to be responsive to the ligand TGFβ^34,35^. The TGFβ pathway model was calibrated using Affymetrix data from A549 lung adenocarcinoma cell line samples stimulated with the ligand TGFβ (Fig. 3A). After freezing the model, biological validation of the TGFβ model was performed on Affymetrix datasets from different cell types. In peripheral blood mononuclear cells, IL-10 (IL-10) induces a tolerogenic state associated with activation of the TGFβ pathway^1,3,36^. The model measured TGFβ activity in IL-10-stimulated primary peripheral blood mononuclear cells (PBMCs) and separated well between control and stimulated samples with the threshold at a lower pathway activity score than in the calibration samples (Fig. 3B). In primary macrophages, measured TGFβ pathway activity increased after stimulation with TGFβ1 with a pathway activity threshold comparable to that in PBMCs (Fig. 3C). In various epithelial cell types, that is, a normal ovarian epithelial cell line, the MDA-MB-231 breast cancer and a TGFβ receptor-expressing LS174T colon cancer cell line, TGFβ pathway activity increased after stimulation with TGFβ1 (Fig. 3D–F). Finally, the model correctly identified induced TGFβ pathway activity in a sample set from mesenchymal stem cells stimulated with TGFβ1 (Fig. 3G). Overall, the model, calibrated on a lung cancer epithelial cell line, clearly distinguished inactive and active samples in various analysed cell types, that is, various epithelial (cancer and non-cancer) and blood cell types and stem cells.

#### NFκB pathway

**Figure 4:**
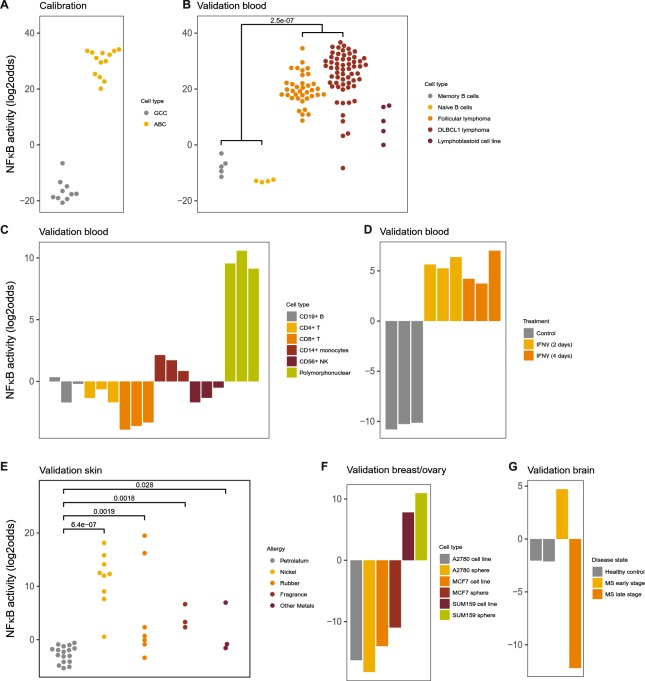
NFκB pathway model. (**A**) Calibration of the NFκB pathway model on dataset GSE12195 containing samples from NFκB-active *activated B cell-like* (ABC) subtype of diffuse large B-cell lymphoma (DLBCL1) and NFκB-inactive germinal centre centroblast (GCC) samples. (**B**–**G**) Validation on GSE12195 sample data that were not used for calibration and on independent GEO datasets, various cell/tissue types. (**B**) GSE12195, healthy B-cells, follicular lymphoma, DLBCL1 with unavailable subtype information, lymphoma cell line. (**C**) GSE72642, peripheral blood cell types, FACS sorted, of healthy individuals. (**D**) GSE58096, THP-1 monocytic cells stimulated with IFNγ or vehicle for indicated time periods. (**E**) GSE60028, patch testing with common antigens (as indicated) and petrolatum (control) was performed on skin of patients with allergic contact dermatitis. (**F**) GSE43657, breast cancer cell lines, among which the SUM159 inflammatory breast cancer cell line, cultured in 2D and 3D (spheroids) setting. (**G**) GSE38010, brain tissue samples from early stage (inflammatory) and late stage (inflammation subsided) multiple sclerosis lesions and healthy controls. The activity score is calculated as log2odds. Two-sided Wilcoxon signed-rank statistical tests were performed; p-values are indicated in the figures.

Twenty-nine NFκB target genes were selected (Supplemental Information). The computational model was calibrated on samples from a subtype of diffuse large B-cell lymphoma (DLBCL1) with a known active NFκB pathway, while a specific B cell type served as NFκB-inactive samples^37^ (Fig. 4A). The calibration dataset contained additional independent validation samples (not used for calibration): healthy memory and naïve B cells were correctly scored as NFκB pathway inactive, while follicular and DBLCL1 lymphomas and lymphoma cell lines showed expected high NFκB pathway activity (Fig. 4B)^37–39^. A few additional validation sets were available. As expected, unstimulated lymphocytes from healthy individuals scored NFκB inactive, while peripheral blood monocytes and polymorphonuclear neutrophils with a constitutively active NFκB pathway scored as active^40,41^ (Fig. 4C). In the monocytic lineage, IFNα is known to activate NFκB in an indirect way via TNFα^42–44^. In THP-1 monocytic cells, NFκB pathway activity increased after stimulation with IFNγ (Fig. 4D). In skin, the NFκB pathway mediates allergic contact dermatitis upon antigen exposure^45^. Indeed, in a dataset with skin tissue samples from patients with allergic contact dermatitis who were exposed to allergenic nickel or non-allergenic petrolatum, the NFκB activity score was very high in nickel-exposed and low in non-allergen-exposed skin samples^46^ (Fig. 4E). Samples in which skin was patched with intermediate allergenic substances showed intermediate NFκB activity scores, demonstrating that the model can measure quantitative differences in NFκB activity (Fig. 4E). Inflammatory breast cancer is NFκB-active^47^. The SUM159 cell line is derived from inflammatory breast cancer and was scored as NFκB active, in contrast to other breast cancer cell lines (Fig. 4F). Finally, multiple sclerosis (MS) is a chronic demyelinating disease of the central nervous system characterized by acute inflammatory events^48^. Inflammatory plaque lesions in the brain have been shown to exhibit an NFκB expression signature, which disappears in the chronic plaque phase^49^. In a small but unique MS dataset, the NFκB pathway score was indeed high in the acute plaque sample and low in healthy brain and chronic plaque samples (Fig. 4G). In conclusion, the NFκB model performed well on a variety of tissue/cell types of blood, epithelial and brain origin.

#### AR pathway

**Figure 5:**
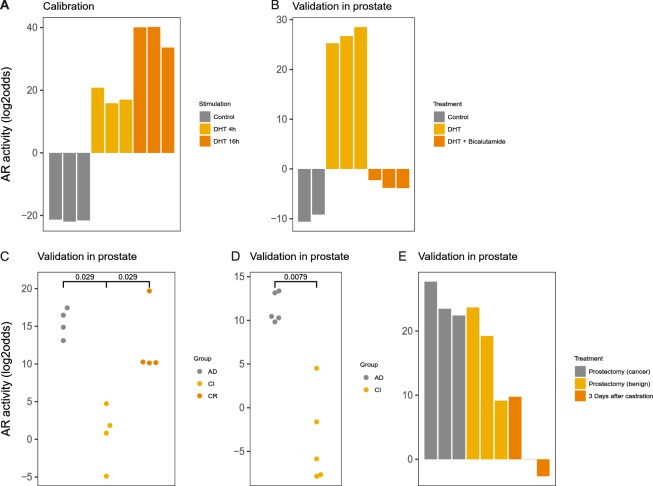
AR pathway model. (**A**) Calibration of the AR pathway model on dataset GSE7868, containing samples from LNCaP prostate cancer cells, treated with 100 nM dihydrotestosterone (DHT) for 0, 4 and 16 h. (**B**–**E**) Validation on independent GEO datasets. (**B**) GSE7708, LNCap cells treated with 1 nM DHT with or without bicalutamide (added 2 h before DHT) for 16 h. (**C**) GSE21887 KUCaP-2 cell grafted in nude mice, androgen-dependent tumour growth (AD), castration-induced tumour regression (CI), and castration-resistant (CR) regrowth. (**D**) GSE33316, LuCaP35 cells grafted in NOD-SCID mice, AD and CI. (**E**) GSE32982, biopsy samples from prostate cancer, benign prostate tissue and prostate cancer tissue three days after surgical castration. The activity score is calculated as log2odds; p-values are indicated in the figures.

Twenty-eight AR target genes were selected (Supplemental information). Prostate cancer is driven by the AR signalling pathway, and an important treatment consists of androgen depletion, e.g., by castration^50^. The model was calibrated using data from AR-expressing LNCaP cell lines stimulated with, or deprived of, the AR-activating ligand dihydrotestosterone (DHT)^51^ (Fig. 5A). The model was frozen and biologically validated. On an independent experiment with the same prostate cancer (PCa) cell line, AR pathway activity increased after DHT treatment and decreased again when DHT was combined with the anti-androgen drug bicalutamide (Fig. 5B). A similarly high AR pathway activity was seen in tumour tissue of mice grafted with a PCa cell line, which decreased after castration (Fig. 5C and D). Finally, patient PCa samples had an active AR pathway, and castration had the same effect as observed in mice (Fig. 5E). In conclusion, the AR pathway model performs as expected on prostate cells/tissue.

#### Clinical validation examples for each of the developed pathway models

**Figure 6:**
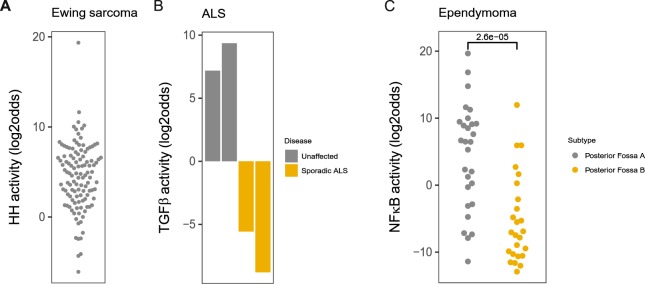
Clinical examples for use of each pathway model. (**A**) Hedgehog pathway, GSE34620. Ewing sarcoma, characterized by expression of the fusion protein EWS-FLI, which activates the Hedgehog transcription factor GLI. (**B**) TGFβ pathway, GSE87385. Patient-derived induced pluripotent stem cell model for amyotrophic lateral sclerosis (ALS), iPS cell lines from 2 ALS patients and 2 healthy individuals were differentiated to oligodendrocytes. (**C**) NFκB pathway, GSE66354. Group A and group B subtypes of ependymoma posterior, group A is the inflammatory phenotype. The activity score is calculated as log2odds. Two-sided Wilcoxon signed-rank statistical tests were performed; p-values are indicated in the figures.

Following biological validation, for each of the pathway models an additional independent Affymetrix dataset from a clinical study was analysed. For the HH pathway model, the rare Ewing sarcoma bone tumour was analysed, characterized by a GLI-activating fusion protein^52,53^. In nearly all patient samples (91%, 107 out of 117 tumour samples), the HH pathway was active as a consequence of the constitutively activated GLI transcription factor (Fig. 6A).

For the TGFβ pathway model, we analysed a small study of amyotrophic lateral sclerosis (ALS), a neurological disease in which demyelination of neurons has been described^54–56^. Functioning of the TGFβ pathway is important for myelinization^57^. To create a cell culture model for ALS, induced pluripotent stem (iPS) cells from healthy individuals and patients with ALS were differentiated to oligodendrocytes, cells that provide metabolic support to neurons and generate myelin sheaths^58^. Oligodendrocytes from healthy persons appeared to have an active TGFβ pathway, while oligodendrocytes from ALS patients were deficient in TGFβ pathway activity (Fig. 6B).

For the NFκB pathway model, we analysed a clinical study on patients with an ependymoma brain tumour. Group A posterior fossa (PF) ependymoma has been associated with an inflammatory profile^59^. Accordingly, the NFκB pathway model scored NFκB activity significantly higher in the majority of patients with PF ependymoma A compared to that in non-inflammatory subtype B (Fig. 6C).

#### Relevance of multiple pathway activity analysis: prostate hyperplasia and cancer

**Figure 7:**
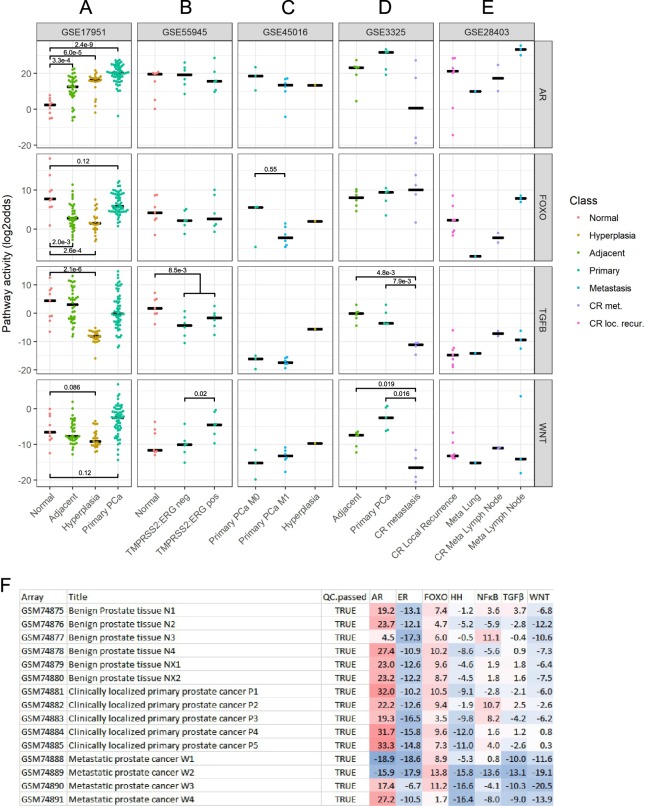
Primary and metastasized prostate cancer as clinical examples for the combined use of multiple pathway analyses. Shown are for the analysed datasets (listed from left to right) from top to bottom: AR, FOXO-PI3K, TGFβ and Wnt pathway activities (indicated at the right side of the figure). Dot plots show the median of the measured pathway scores. (**A**) GSE17951, macrodissected tissue samples from normal (healthy) prostate (n = 9), cancer-adjacent prostate (n = 36), hyperplasia (n = 23) and primary prostate cancer (n = 57). (**B**) GSE55945, macrodissected tissue samples from “normal” (unknown whether healthy or cancer-adjacent) prostate (n = 7) and low-grade primary prostate cancer (Gleason score 6–7), two subtypes characterized by absence (middle, n = 6) and presence (right, n = 6) of the fusion gene TMPRSS2:ERG. Statistics: one-sided Wilcoxon rank-sum test to compare fusion-positive (known Wnt active) and fusion-negative group. (**C**) GSE45016, microdissected tissue samples from high-grade (Gleason 8–9) primary prostate cancer stage M0 without metastases (n = 3) and M1 with metastasis (n = 6) and hyperplasia (n = 1). (**D**) GSE3325, macrodissected tissue samples from cancer-adjacent prostate (n = 6), primary cancer (n = 5), and castrate-resistant (CR) metastases (n = 4); Statistics: one-sided Wilcoxon rank-sum test to compare TGFβ pathway activities, known TGFβ loss in PCa. (**E**) GSE28403, CR local recurrence (n = 7), metastasis lung (n = 1), CR lymph node metastasis (n = 2), metastasis lymph node (n = 3). The activity score is calculated as log2odds. P-values are indicated in the figures. (**F**) GSE3325, iIlustrative presentation of individual patient samples with multiple pathway scores. From left to right: GSM number indicating sample number; clinical annotation as supplied in GEO, ‘benign’ indicates benign tissue adjacent to cancer; Quality Control (TRUE means: passed QC); individual pathway activity scores indicated as log2odds with colour coding ranging from blue (most inactive) to red (most active). Unless otherwise indicated, two-sided Wilcoxon signed-rank statistical tests were performed; p-values are indicated in the figures.

To illustrate clinical relevance of measuring multiple pathway activities, in Affymetrix datasets from clinical prostate studies, ER, AR, PI3K, Wnt, TGFβ, HH and NFκB pathway activities were analysed (Fig. 7). Individual ER, HH and NFκB pathway activity results did not provide distinctive information. Except for dataset GSE17951, sample sizes were small, and differences in sample preparation (as indicated in figure legend) precluded comparison across datasets, limiting statistical analysis. However, since they contained rare samples of advanced disease and for completeness sake, we chose to analyse and present all available studies. GSE17951 contained samples from healthy and cancer-adjacent prostate, benign hyperplasia, and primary PCa (Fig. 7A). Compared to healthy tissue, AR pathway activity was increased in all three pathological conditions. Prostate hyperplasia showed a pathway activity profile that was distinct from PCa and characterized by reduced FOXO activity, indicating PI3K pathway activity and loss of TGFbeta and Wnt pathway activity. In contrast, in primary PCa, the Wnt pathway showed a trend towards increased activity (p = 0.12), and median FOXO activity was only slightly lower than in healthy tissue (p = 0.12) indicating that PI3K pathway activity was restricted to a subgroup of PCa. GSE55945 contained ‘normal’ (non-cancer) prostate samples and low-grade primary PCa characterized by the absence or presence of the TMPRSS2:ERG fusion gene (Fig. 7B). TGFβ activity was lower in PCa compared to that in non-cancer ‘normal’ tissue. In fusion gene-positive PCa, Wnt pathway activity was increased. GSE45016 contained samples from high-grade primary PCa without (M0) or with (M1) metastasis and one hyperplasia sample (Fig. 7C). In comparison with M0, M1 samples had lower FOXO activity, indicating PI3K pathway activity. TGFβ and Wnt activity were completely lost in these high grade tumours. GSE3325 contained cancer-adjacent, primary PCa, and castrate-resistant (CR) metastatic tissue samples (Fig. 7D). In CR metastatic tissue, some distinct features were observed: median AR pathway activity was low but highly variable, indicating that this group also contained samples with high AR activity, and TGFβ and Wnt pathway activity were lost. GSE28403 contained a small number of samples from advanced PCa patients with varying PI3K pathway activity and loss of TGFβ and Wnt activity (Fig. 7E).

In summary, AR pathway activity was increased in cancer-adjacent, hyperplasia and PCa tissue and was nearly always associated with PI3K pathway activity in hyperplasia and variably in primary PCa. Wnt pathway activity was increased in TMPRSS2:ERG fusion gene-positive primary PCa but, together with TGFβ pathway activity, was frequently lost in advanced and CR disease. In CR PCa, both AR and PI3K pathway activity were highly variable.

To enable appreciation of analysing multiple pathway activities *per individual patient sample*, as an illustration, pathway activity scores are shown *per patient* for GSE3325 (Fig. 7F). Specific pathway combinations can be clinically relevant, for example, by providing information on potential resistance pathways. A significant inverse correlation between AR and NFκB pathway activity was observed in cancer-adjacent tissue (Pearson −0.9; p = 0.019) (see Supplemental information Fig. 2A), and in the small primary PCa group, a similar trend was observed (Pearson −0.8, p = 0.10). This inverse relationship could be confirmed in the large GSE17951 dataset (Fig. 7A) in cancer-adjacent (Pearson −0.6, p = 0.0004), benign hyperplasia (Pearson −0.6, p = 0.006), and primary PCa tissue (Pearson −0.4, p = 0.004) but was not present in healthy prostate tissue (Pearson 0.01, p = 0.97; Supplemental information Fig. 2C). Interestingly, in one of the CR metastatic tumours, complete loss of AR pathway activity was associated with a relatively high NFκB score, which fits an inverse relationship between these pathways (Fig. 7F, sample GSM74888).

## Discussion

Over the past decade, we have developed a new method enabling quantitative measurement of activity of the major signal transduction pathways in a wide variety of cell and tissue types based on computational inference of pathway activity from measurements of *mRNA levels of well-validated direct target genes of a transcription factor* associated with the respective signalling pathway^6–8^. While the mRNA level of an individual target gene of a signalling pathway is not a reliable marker for pathway activity, we provided evidence that measuring a set of typically 20−35 target mRNAs enables highly sensitive and specific analysis of pathway activity. The set of target genes for each model, i.e., for each transcription factor, is carefully selected based on combined experimental evidence for these genes being (preferably direct) target genes, preferably shown in various cell types and by multiple research groups that are expert in a certain signalling pathway. Although in principle target gene mRNA levels, serving as input for the computational pathway models, can be measured using any modality (like qPCR or RNA sequencing), we have in the first instance chosen Affymetrix HG-U133Plus2.0 microarrays for model calibration and validation purposes because of publicly available preclinical and clinical study data in repositories such as GEO and because processing is standardized. For calibration and biological validation (after freezing the models) of TGFβ, HH, AR and NFκB pathway models, Affymetrix datasets were selected containing data from samples with a ‘ground truth’ pathway activity. For TGFβ and Hedgehog pathway models, epithelial calibration datasets were chosen; subsequently, the models also performed well on brain, blood and bone-derived samples. The NFκB pathway model was calibrated on blood samples and worked equally well when used on epithelial and brain cell types. For the AR pathway model, prostate tissue was the only available cell type for validation purposes. In conclusion, biological validation results were successfully obtained for these pathway models, including on sample data from patients with various diseases, i.e., for the NFκB pathway model allergic contact dermatitis, multiple sclerosis, and lymphomas, for the HH pathway BCC and medulloblastoma, and for the AR pathway PCa.

One reason for applicability of pathway models without model adaptation on multiple cell types and irrespective of disease type is that pathway target genes were chosen preferentially as *direct* target genes of the transcription factor, meaning minimal involvement of cell type-specific proteins, to reduce cell type-specific influences on target gene expression. Also important is that target mRNAs were not selected based on relevance for a specific disease, e.g., cancer or tissue type. Last but not least, Bayesian network models can deal well with variability in input data, including conflicting data such as occasional target genes that are not expressed in a specific sample despite the transcription factor being active, or target genes that are expressed despite an inactive transcription factor. Consequently, when expression levels of individual pathway target genes vary between samples, for example, between different cell types, the Bayesian reasoning principle still allows robust interpretation of these mRNA levels. This likely also explains why the pathway models can deliver reliable pathway activity measurements across patient samples despite the variation that is inherent to such samples. Even when minimal/maximal pathway activity scores vary across cell types, as illustrated by HH and TGFβ pathway model validation results, the models remained capable of discriminating between low and high pathway activity without adapting model parameters.

We believe that the pathway models can also be used on cell/tissue types for which no validation results were presented on the premise that a few reference samples from the respective cell type are available with ‘ground truth’ pathway activity. Even without this, comparison between a pathway activity score in normal and disease tissue of a single patient may provide information with respect to potential pathogenic pathway activity.

A number of other RNA-based pathway analysis tools are available, such as Gene Set Enrichment Analysis (GSEA) and DAVID, using pathway information from databases such as KEGG (www.kegg.jp) and WikiPathways (www.wikipathways.org), which use the denomination ‘pathways’ not only for signal transduction pathways but also for various other intracellular mechanisms^60,61^. These methods are however not intended for measuring pathway activity in a single test sample, which is a crucial requirement for diagnostic use and forms the core of the approach described here. Their use lies in discovering which ‘pathways’ differ between two (or more) groups of samples that are compared in a data-driven manner. Furthermore, they do not define a rational biological measure of activation based on signalling biology like we present here. Instead, they are based on algebraic notions such as overrepresentation of a set of differentially expressed genes in a pathway list or rankings of a pathway’s gene set in an ordered list of differentially expressed genes between the groups to compare, while differences between mRNA, protein and activated protein levels are not taken into account. As far as validation is concerned, these other methods usually report a p-value to indicate how likely it would have been to obtain the reported results on random(ized) data, but as far as we know, they have not been biologically validated on sample sets with ‘ground truth’ pathway activity data. Such a true biological validation is important to ensure correct assessment of functional activity of signalling pathways in an individual patient sample prior to use for diagnostic applications. For illustration purpose, we have compared our pathway analysis approach with GSEA on a sufficiently large dataset with solid ‘ground truth’ information, consisting of 32 colon adenoma samples and 32 matched normal colon samples (GSE8671) (Supplemental Information: Comparison GSEA with pathway analysis). In colon adenoma the Wnt pathway is the dominant active signalling pathway^62,63^. Our analysis of this dataset confirmed the Wnt pathway as the most active pathway in the adenoma tissue samples, with the PI3K pathway as a second active pathway^6,8^. In comparison, GSEA analysis listed the first Wnt-related pathway at rank 87 of listed ‘pathways’ upregulated in the adenoma versus control tissue group, with a false-discovery rate corrected p-value of 0.038, while other Wnt-related pathways were not significantly upregulated. In contrast, many other pathways were listed in the top 10 up- or downregulated pathways without any clear biological relation to colon adenomas versus normal colon.

To illustrate potential diagnostic value of a pathway model-based test for a clinical or preclinical research question, example clinical datasets were analysed. For the Hedgehog pathway, we analysed a dataset from a clinical study containing a large number of patient samples from Ewing sarcoma^64^ and found the Hedgehog pathway to be nearly uniformly active. This is in agreement with Ewing sarcoma being characterized by expression of the EWSR1-FLI1 Ewing fusion gene, known to result in activation of the GLI transcription factor in the HH pathway and therefore in an active HH pathway^52,53^. Using this result as initial proof that the HH model correctly reads out pathway activity in bone tissue, it enables exploration of the role of HH pathway activity not only in Ewing sarcoma but also in osteosarcoma and investigation of the use of the HH pathway model to identify patients that may benefit from anti-HH drugs such as arsenic trioxide (ATO), intraconazole, and vismodegib^65^.

For the TGFβ pathway model, we analysed a small study in which an elaborate *in vitro* cell culture model was generated for amyotrophic lateral sclerosis (ALS). ALS is a neurodegenerative disease for which no treatment exists, with largely unknown pathophysiology, in which demyelination of neurons is thought to play a pathogenic role^54,66^. In this study, iPS cell lines were generated from patients with ALS and healthy individuals and were differentiated to oligodendrocytes as an experimental model system for ALS^67^. TGFβ pathway activity in oligodendrocytes is important for myelination of neurons^56,58^. Pathway analysis revealed a remarkable loss of TGFβ pathway activity in oligodendrocytes from ALS patients, suggesting a pathogenic role for loss of TGFβ pathway activity in this disease. This finding is in full agreement with reports describing loss-of-function mutations in the ZNF512B gene that result in loss of TGFβ pathway activity and are associated with susceptibility to, and more aggressive, ALS^68,69^. Recently, an elegant mouse model revealed that loss of TGFβ pathway activity in glia cells causes demyelinating diseases^70^. In an ALS mouse model, treatment with a TGFβ-activating drug had a therapeutic effect^71^. Thus, although only a few samples were available for analysis, the results support existing evidence on a role for reduced TGFβ activity in ALS and are expected to provide a means to measure TGFβ pathway activity in brain tissue samples of ALS patients. This example illustrates how signalling pathway models can be used as informative readout of preclinical disease models, for example, to identify new drug targets and screen for compounds to restore the pathway defect, even if only a few samples are available.

Inflammatory processes play a role in many diseases and are associated with NFκB pathway activation. Group A subtype of posterior fossa ependymoma, a rare paediatric brain tumour, has been characterized by an inflammatory expression profile and has a higher mortality rate than those of the Group B subtype^72^. As an illustrative clinical example of NFκB pathway activity, analysis of a series of ependymomas showed high incidence of NFκB activation in Group A subtype, which not only provides further proof for use of the NFκB model in brain tissue samples but also supports a pathogenic role for this pathway in this subtype and a potential new avenue for personalized targeted treatment.

Signalling pathways often interact, simultaneously or sequentially, to coordinate cellular processes, and analysis of multiple pathways enables a more comprehensive characterization of disease pathophysiology. We selected PCa, with AR pathway activity as its hallmark, as a clinical example to illustrate the value of measuring more than one signalling pathway, including analysis of Wnt and PI3K pathway activity. In addition to expected increased AR pathway activity in primary PCa, AR pathway activity was also increased in cancer-adjacent and hyperplastic prostate tissue, which is likely caused by elevated local androgen levels^73,74^. In contrast to primary PCa, AR pathway activity was highly variable in CR disease, probably caused by variations in local production of androgens or the presence of activating mutations in the androgen receptor^75,76^. The PI3K pathway seemed to be activated only in a subpopulation of primary PCa while frequently highly active in advanced PCa. The latter is in agreement with its reported role in metastatic and castrate-resistant prostate cancer^38,77,78^. In primary PCa, it is conceivable that a PI3K pathway activating mutation, or loss of PTEN, drives only a subgroup of cancers, possibly conferring a bad prognosis. Indeed, the presence of a putative protein signature for PI3K pathway activity, consisting of PTEN/pAKT/pS6/stathmin immunohistochemistry staining, was shown to correlate with bad outcome of PCa^79^. Developmental pathways, such as the TGFβ and Wnt pathways, can play tumour-suppressive and tumour-promoting roles in cancer^80^. TGFβ pathway activity was frequently reduced or lost, especially in advanced PCa, indicating loss of its tumour suppressive activity, which is a well-known phenomenon in prostate cancer^81–83^. One possible mechanism behind this observation may be a direct repressive effect of AR on the SMAD transcription factor of the TGFβ pathway^84^. The role of Wnt pathway activity in prostate cancer is complex^85,86^. In primary PCa, this pathway was frequently activated, and separate analysis of TMPRSS2:ERG fusion gene-negative and -positive cancers revealed that the presence of this fusion protein was the most likely cause of Wnt pathway activity. Indeed, TMPRSS2:ERG fusion is frequently present in prostate cancer and known to activate the Wnt pathway through overproduction of the ERG protein, fully in line with our findings^87^. This result provides additional evidence that our model can correctly measure Wnt pathway activity in prostate tissue and confirms the presumed tumour-promoting role of the Wnt pathway^85,86^. However, in advanced disease, Wnt pathway activity is generally lost, suggesting a tumour-suppressive rather than tumour-promoting role under this condition. In contrast to primary PCa, in prostate hyperplasia a consistent loss of Wnt and TGFβ pathway activity associated with PI3K pathway activity was found, suggesting a distinct pathophysiology with a putative tumour-suppressor role for both Wnt and TGFβ pathways. The frequent PI3K pathway activity, characterized by reduced FOXO activity, resembles that found in benign colon adenomas^6^. Furthermore, in prostate tissue, FOXO activity has been described to confer a tumour-suppressor role to a co-active Wnt pathway, quite similar to what has been reported for combined FOXO-TGFβ pathway activity in breast tissue^8,88,89^. This suggests that PI3K pathway activity may interfere with tumour-suppressive functions of Wnt and TGFβ pathways by eliminating FOXO activity. Clearly, delineating the role of the Wnt pathway in various forms of prostate disease requires more investigation, and the described pathway model may be of help in standardizing pathway measurements.

Measuring multiple pathways in individual samples revealed potential mechanisms of drug resistance. Although measured loss of AR activity in CR disease provides an obvious reason for anti-androgen therapy resistance, NFκB and PI3K pathway activities may also cause hormonal resistance^90,91^. The inverse relation between AR and NFκB pathway activity in cancer-adjacent tissue, hyperplasia, and primary PCa is of potential clinical relevance since inflammation is known to be a bad prognostic marker for both benign hyperplasia and prostate cancer and may be associated with hormonal resistance^92–94^. The underlying mechanism is likely the negative interaction between NFκB and AR transcription factors^95^. Whether this can be extended to advanced cancer could not be investigated due to small sample numbers; however, the one CR tumour with inactive AR pathway and active NFκB pathway is illustrative of a similar relationship where NFκB activity may play a role in castrate resistance^95^. Taken together, pathway activity results in prostate hyperplasia and cancer were in agreement with expectations based on the literature, providing further validation of the used pathway analysis models. We realize this is only an explorative study; however, upon confirmation of our findings, signalling pathway measurements in patient samples may be of value to predict response to targeted drugs including PI3K inhibitors, for example, in patients with advanced and CR prostate cancer, and provide information on potential (targetable) resistance pathways. Another clinical utility could be quantitative monitoring of AR pathway activity to support bipolar androgen therapy (BAT), an approach in which androgen and anti-androgen therapy are sequentially used^96^.

In summary, following biological validation, Bayesian network models for measurement of cellular signal transduction pathway activity were used to analyse sample data of a variety of clinical disorders. We illustrated how this new approach can be used to quantitatively characterize disease pathophysiology with a focus on prostate cancer. Importantly, the described pathway analysis is envisioned to provide clinically actionable results for individual patients, for example, prediction of response and resistance to targeted drugs, providing targets to reverse drug resistance, and monitoring of therapy response. Following extended clinical validation, a major future application will be in diagnostics, aiming at optimizing (targeted) treatment for individual patients. Many additional applications are possible, for example, in cell and tissue culture, regenerative medicine, toxicology, and drug development (target discovery, compound screening and lead optimization)^97,98^. To enable application to clinical routine formalin-fixed paraffin-embedded (FFPE) tissue samples, pathway models are being translated from Affymetrix microarray to qPCR and RNA sequencing as input. Simultaneously, multiple clinical studies for clinical validation are in progress.

## Supplementary information

Supplemental Information
