# Publisher Correction: Enabling precision medicine by unravelling disease pathophysiology: quantifying signal transduction pathway activity across cell and tissue types

**DOI:** 10.1038/s41598-020-60886-7

**Published:** 2020-03-04

**Authors:** Anja van de Stolpe, Laurent Holtzer, Henk van Ooijen, Marcia Alves de Inda, Wim Verhaegh

**Affiliations:** Philips Research, High Tech Campus 11, 5656 AE Eindhoven, The Netherlands

Correction to: *Scientific Reports* 10.1038/s41598-018-38179-x, published online 07 February 2019

In the original version of this Article, the authors Anja van de Stolpe and Marcia Alves de Inda were incorrectly indexed. These errors have now been corrected.

