# Supplementary material for: Enabling precision medicine by unravelling disease pathophysiology: quantifying signal transduction pathway activity across cell and tissue types
Source: Sci Rep. 2019 Feb 7;9:1603. doi: 10.1038/s41598-018-38179-x (PMC6367506; doi:10.1038/s41598-018-38179-x)
Supplement: Supplementary file 1 — Supplemental Information [file 41598_2018_38179_MOESM1_ESM.pdf]

SUPPLEMENT

Enabling precision medicine by unravelling disease pathophysiology: quantifying signal transduction pathway activity across cell and tissue types

Anja van de Stolpe, Laurent Holtzer, Henk van Ooijen, Marcia Alves de Inda, Wim Verhaegh

Philips Research, Eindhoven, The Netherlands

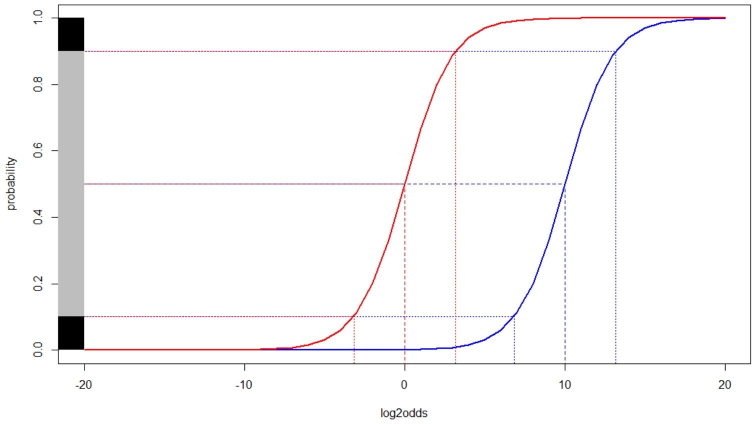

Supplement Figure 1. The relationship between Pathway Activity score, calculated as *lod2odds* (x-axis) and *probability* (y-axis). The direct output of the model calculation is the probability (p) that the transcription factor is active; this can be used to calculate the odds {odds=p/(1-p)} and the log2odds pathway score.

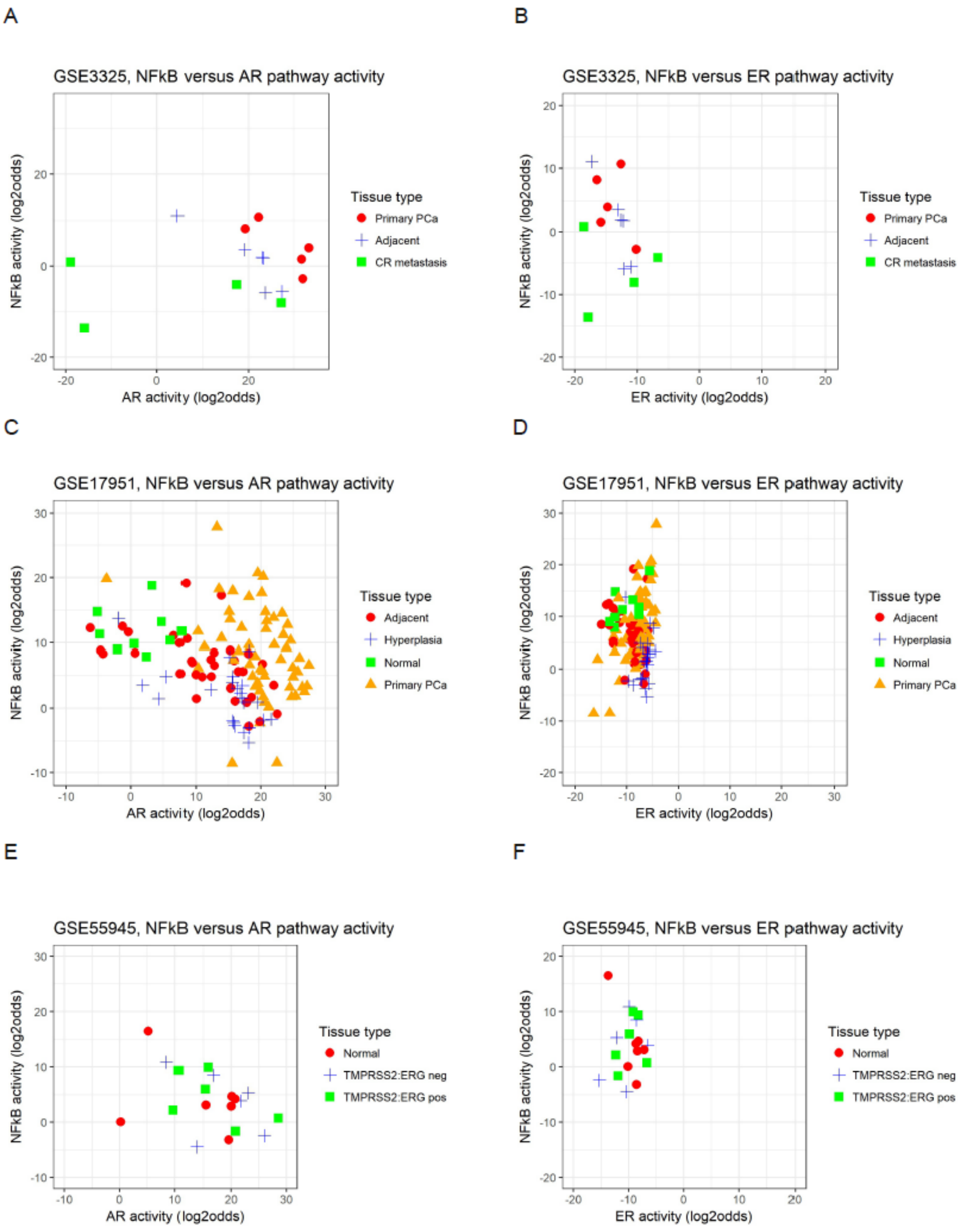

Supplement Figure 2.

Correlation plots for NFkB pathway activity versus AR pathway activity (A,C,E) and ER pathway activity (B,D,F) in cancer-adjacent tissue, benign hyperplasia and primary prostate cancer samples from GEO datasets GSE3325, GSE17951, GSE55945

Supplement Table 1

QC results on GEO datasets

Dataset	Figure	# of samples analyzed	# QC passed	# QC failed	ID QC failed samples
GSE7553	1A	19	19	0	
GSE39612	1B	66	66	0	
GSE29316	1C	6	6	0	
GSE37418	1D	76	61	15	GSM918579, GSM918580, GSM918581, GSM918585, GSM918599, GSM918617, GSM918623, GSM918628, GSM918631, GSM918632, GSM918635, GSM918648, GSM918649, GSM918652, GSM918653
GSE49243	1E	73	71	2	GSM1195809, GSM1195844
GSE17708	2A	6	6	0	
GSE43700	2B	8	8	0	
GSE7568	2C	15	15	0	
GSE6653	2D	8	8	0	
GSE14491	2E	16	16	0	
GSE59771	2F	4	4	0	
GSE84500	2G	24	24	0	
GSE12195	3A	23	23	0	
GSE12195	3B	113	111	2	GSM476262, GSM476276
GSE72642	3C	18	18	0	
GSE58096	3D	9	9	0	
GSE60028	3E	47	39	8	GSM1463949, GSM1463953, GSM1463962, GSM1463966, GSM1463974, GSM1463981, GSM1463983, GSM1463984
GSE43657	3F	6	6	0	
GSE38010	3G	5	4	1	GSM931818
GSE7868	4A	9	9	0	
GSE7708	4B	8	8	0	
GSE21887	4C	12	12	0	
GSE33316	4D	10	10	0	
GSE32982	4E	9	9	0	
GSE34620	5A	117	115	2	GSM852014, GSM852036
GSE87385	5B	4	4	0	
GSE66354	5C	55	53	2	GSM1620217, GSM1620239
GSE17951	6A	154	125	29	GSM449148, GSM449211, GSM449261, GSM449266, GSM449278, GSM449256, GSM449258, GSM449270, GSM449273, GSM449275, GSM449281, GSM449286, GSM449287, GSM449288, GSM449238, GSM449242, GSM449295, GSM449300, GSM449175, GSM449180, GSM449221, GSM449229, GSM449231, GSM449232, GSM449233, GSM449237, GSM449248, GSM449263, GSM449265
GSE55945	6B	19	19	0	
GSE45016	6C	11	10	1	GSM1095880
GSE3325	6D/6F	15	15	0	
GSE28403	6E	13	13	0	

Supplement List with GEO datasets used.

Numbers/GEO links of datasets from the GEO database that were used for model calibration and validation purposes, and associated publications.

Figure 2

GSE7553, https://www.ncbi.nlm.nih.gov/geo/query/acc.cgi?acc=GSE7553. Riker AI, Enkemann SA, Fodstad O, Liu S, Ren S, Morris C, Xi Y, Howell P, Metge B, Samant RS, Shevde LA, Li W, Eschrich S, Daud A, Ju J, Matta J. The gene expression profiles of primary and metastatic melanoma yields a transition point of tumor progression and metastasis. BMC Med Genomics. 2008 Apr 28;1:13.

GSE39612, https://www.ncbi.nlm.nih.gov/geo/query/acc.cgi?acc=GSE39612. Harms PW, Patel RM, Verhaegen ME, Giordano TJ, Nash KT, Johnson CN, Daignault S, Thomas DG, Gudjonsson JE, Elder JT, Dlugosz AA, Johnson TM, Fullen DR, Bichakjian CK. Distinct gene expression profiles of viral- and nonviral-associated merkel cell carcinoma revealed by transcriptome analysis. J Invest Dermatol. 2013 Apr;133(4):936-45.

GSE29316, https://www.ncbi.nlm.nih.gov/geo/query/acc.cgi?acc=GSE29316. Chen W, Tang T, Eastham-Anderson J, Dunlap D et al. Canonical hedgehog signaling augments tumor angiogenesis by induction of VEGF-A in stromal perivascular cells. Proc Natl Acad Sci U S A 2011 Jun 7;108(23):9589-94

GSE49243, https://www.ncbi.nlm.nih.gov/geo/query/acc.cgi?acc=GSE49243. Pöschl J, Stark S, Neumann P, Gröbner S et al. Genomic and transcriptomic analyses match medulloblastoma mouse models to their human counterparts. Acta Neuropathol 2014 Jul;128(1):123-36

GSE37418, https://www.ncbi.nlm.nih.gov/geo/query/acc.cgi?acc=GSE37418. GSE49243, https://www.ncbi.nlm.nih.gov/geo/query/acc.cgi?acc=GSE49243. Robinson G, Parker M, Kranenburg TA, Lu C et al. Novel mutations target distinct subgroups of medulloblastoma. Nature 2012 Aug 2;488(7409):43-8

Figure 3

GSE17708, https://www.ncbi.nlm.nih.gov/geo/query/acc.cgi?acc=GSE17708. Sartor MA, Mahavisno V, Keshamouni VG, Cavalcoli J et al. ConceptGen: a gene set enrichment and gene set relation mapping tool. Bioinformatics 2010 Feb 15;26(4):456-63.

GSE43700, https://www.ncbi.nlm.nih.gov/geo/query/acc.cgi?acc=GSE43700. Teles RM, Graeber TG, Krutzik SR, Montoya D et al. Type I interferon suppresses type II interferon-triggered human anti-mycobacterial responses. Science 2013 Mar 22;339(6126):1448-53

GSE7568, https://www.ncbi.nlm.nih.gov/geo/query/acc.cgi?acc=GSE7568. Gratchev A, Kzhyshkowska J, Kannookadan S, Ochsenreiter M et al. Activation of a TGF-beta-specific multistep gene expression program in mature macrophages requires glucocorticoid-mediated surface expression of TGF-beta receptor II. J Immunol 2008 May 15;180(10):6553-65

GSE6653, https://www.ncbi.nlm.nih.gov/geo/query/acc.cgi?acc=GSE6653. Qin H, Chan MW, Liyanarachchi S, Balch C et al. An integrative ChIP-chip and gene expression profiling to model SMAD regulatory modules. BMC Syst Biol 2009 Jul 17;3:73

GSE14491, https://www.ncbi.nlm.nih.gov/geo/query/acc.cgi?acc=GSE14491. Adorno M, Cordenonsi M, Montagner M, Dupont S et al. A Mutant-p53/Smad complex opposes p63 to empower TGFbeta-induced metastasis. Cell 2009 Apr 3;137(1):87-98

GSE59771, https://www.ncbi.nlm.nih.gov/geo/query/acc.cgi?acc=GSE59771. No associated publication available

GSE84500, https://www.ncbi.nlm.nih.gov/geo/query/acc.cgi?acc=GSE84500. van Zoelen EJ, Duarte I, Hendriks JM, van der Woning SP. TGFβ-induced switch from adipogenic to osteogenic differentiation of human mesenchymal stem cells: identification of drug targets for prevention of fat cell differentiation. Stem Cell Res Ther 2016 Aug 26;7(1):123

Figure 4

GSE12195, https://www.ncbi.nlm.nih.gov/geo/query/acc.cgi?acc=GSE12195. Compagno M, Lim WK, Grunn A, Nandula SV et al. Mutations of multiple genes cause deregulation of NF-kappaB in diffuse large B-cell lymphoma. Nature 2009 Jun 4;459(7247):717-21.

GSE72642, https://www.ncbi.nlm.nih.gov/geo/query/acc.cgi?acc=GSE72642. Du X, Tang Y, Xu H, Lit L et al. Genomic profiles for human peripheral blood T cells, B cells, natural killer cells, monocytes, and polymorphonuclear cells: comparisons to ischemic stroke, migraine, and Tourette syndrome. Genomics 2006 Jun;87(6):693-703

GSE58096, https://www.ncbi.nlm.nih.gov/geo/query/acc.cgi?acc=GSE58096. Leu JS, Chen ML, Chang SY, Yu SL et al. SP110b Controls Host Immunity and Susceptibility to Tuberculosis. *Am J Respir Crit Care Med* 2017 Feb 1;195(3):369-382.

GSE60028, https://www.ncbi.nlm.nih.gov/geo/query/acc.cgi?acc=GSE60028. Dhingra N, Shemer A, Correa da Rosa J, Rozenblit M, Fuentes-Duculan J, Gittler JK, Finney R, Czarnowicki T, Zheng X, Xu H, Estrada YD, Cardinale

I, Suárez-Fariñas M, Krueger JG, Guttman-Yassky E. Molecular profiling of contact dermatitis skin identifies allergen-dependent differences in immune response. J Allergy Clin Immunol. 2014 Aug;134(2):362-72)
GSE43657, https://www.ncbi.nlm.nih.gov/geo/query/acc.cgi?acc=GSE43657. Wang H, Zhang Y, Du Y. Ovarian and breast cancer spheres are similar in transcriptomic features and sensitive to fenretinide. Biomed Res Int 2013;2013:510905
GSE38010, https://www.ncbi.nlm.nih.gov/geo/query/acc.cgi?acc=GSE38010. Han MH, Lundgren DH, Jaiswal S, Chao M et al. Janus-like opposing roles of CD47 in autoimmune brain inflammation in humans and mice. *J Exp Med* 2012 Jul 2;209(7):1325-34.

Figure 5
GSE7868, https://www.ncbi.nlm.nih.gov/geo/query/acc.cgi?acc=GSE7868. Wang Q, Li W, Liu XS, Carroll JS et al. A hierarchical network of transcription factors governs androgen receptor-dependent prostate cancer growth. Mol Cell 2007 Aug 3;27(3):380-92.
GSE7708, https://www.ncbi.nlm.nih.gov/geo/query/acc.cgi?acc=GSE7708. Nickols NG, Dervan PB. Suppression of androgen receptor-mediated gene expression by a sequence-specific DNA-binding polyamide. Proc Natl Acad Sci U S A 2007 Jun 19;104(25):10418-23.
GSE21887, https://www.ncbi.nlm.nih.gov/geo/query/acc.cgi?acc=GSE21887. Terada N, Shimizu Y, Kamba T, Inoue T et al. Identification of EP4 as a potential target for the treatment of castration-resistant prostate cancer using a novel xenograft model. Cancer Res 2010 Feb 15;70(4):1606-15.
GSE33316, https://www.ncbi.nlm.nih.gov/geo/query/acc.cgi?acc=GSE33316. Sun Y, Wang BE, Leong KG, Yue P et al. Androgen deprivation causes epithelial-mesenchymal transition in the prostate: implications for androgen-deprivation therapy. Cancer Res 2012 Jan 15;72(2):527-36
GSE32982, https://www.ncbi.nlm.nih.gov/geo/query/acc.cgi?acc=GSE32982. Vaarala MH, Hirvikoski P, Kauppila S, Paavonen TK. Identification of androgen-regulated genes in human prostate. Mol Med Rep 2012 Sep;6(3):466-72.

Figure 6
GSE34620, https://www.ncbi.nlm.nih.gov/geo/query/acc.cgi?acc=GSE34620. Postel-Vinay S, Véron AS, Tirode F, Pierron G et al. Common variants near TARDBP and EGR2 are associated with susceptibility to Ewing sarcoma. Nat Genet 2012 Feb 12;44(3):323-7
GSE87385, https://www.ncbi.nlm.nih.gov/geo/query/acc.cgi?acc=GSE87385. Ferraiuolo L, Meyer K, Sherwood TW, Vick J et al. Oligodendrocytes contribute to motor neuron death in ALS via SOD1-dependent mechanism. Proc Natl Acad Sci U S A 2016 Oct 18;113(42):E6496-E6505
GSE66354, https://www.ncbi.nlm.nih.gov/geo/query/acc.cgi?acc=GSE66354. Griesinger AM, Josephson RJ, Donson AM, Mulcahy Levy JM et al. Interleukin-6/STAT3 Pathway Signaling Drives an Inflammatory Phenotype in Group A Ependymoma. Cancer Immunol Res 2015 Oct;3(10):1165-74.

Figure 7
GSE17951, https://www.ncbi.nlm.nih.gov/geo/query/acc.cgi?acc=GSE17951. Wang Y, Xia XQ, Jia Z, Sawyers A et al. In silico estimates of tissue components in surgical samples based on expression profiling data. Cancer Res 2010 Aug 15;70(16):6448-55.
GSE55945, https://www.ncbi.nlm.nih.gov/geo/query/acc.cgi?acc=GSE55945. Arredouani MS, Lu B, Bhasin M, Eljanne M et al. Identification of the transcription factor single-minded homologue 2 as a potential biomarker and immunotherapy target in prostate cancer. Clin Cancer Res 2009 Sep 15;15(18):5794-802
GSE45016, https://www.ncbi.nlm.nih.gov/geo/query/acc.cgi?acc=GSE45016. Satake H, Tamura K, Furihata M, Anchi T et al. The ubiquitin-like molecule interferon-stimulated gene 15 is overexpressed in human prostate cancer. Oncol Rep 2010 Jan;23(1):11-6
GSE28403, https://www.ncbi.nlm.nih.gov/geo/query/acc.cgi?acc=GSE28403. Vainio P, Wolf M, Edgren H, He T et al. Integrative genomic, transcriptomic, and RNAi analysis indicates a potential oncogenic role for FAM110B in castration-resistant prostate cancer. Prostate 2012 May 15;72(7):789-802
GSE3325, https://www.ncbi.nlm.nih.gov/geo/query/acc.cgi?acc=GSE3325. Varambally S, Yu J, Laxman B, Rhodes DR et al. Integrative genomic and proteomic analysis of prostate cancer reveals signatures of metastatic progression. Cancer Cell 2005 Nov;8(5):393-406

Supplemental Reference-based selection of pathway model target genes

Pathway target gene selection

Potential target genes were selected based on literature (PubMed), and scored according to available target gene evidence:

1. The gene promoter/enhancer region contains a response (enhancer) element motif.
2. The pathway-associated trancription factor (TF) (differentially) binds to the promoter/enhancer region of the gene in question, e.g. demonstrated by EMSA (electrophoretic mobility shift assay) or ChIP (chromatin immunoprecipitation).
3. The enhancer/promoter binding motif is proven to be functional, e.g., by means of a transient transfection assay in which the specific motif is linked to a reporter gene.
4. The gene is differentially transcribed when the pathway is active, demonstrated by for example fold enrichment of the mRNA of the gene as measured by real time PCR, or expression microarray.
5. The gene is differentially transcribed when the pathway is active in the presence of cyloheximide (preventing indirect translated protein-mediated effects)
6. Transcriptional regulation of the target gene is as specific as possible for the pathway of interest.

AR, TGFβ, HH, and NFkB pathway target gene selection, Affymetrix U133 Plus2.0 probeset lists, references for target gene selection

1A. AR pathway target gene probeset list

Gene	Probeset	Gene	Probeset
ABCC4	1554918_a_at	KLK3	204582_s_at
	1555039_a_at		204583_x_at
	203196_at	LCP1	208885_at
APP	200602_at	LRIG1	211596_s_at
	211277_x_at		238339_x_at
	214953_s_at	NDRG1	200632_s_at
AR	211110_s_at	NKX3-1	209706_at
	211621_at		211497_x_at
	226192_at		211498_s_at
CDKN1A	202284_s_at	NTS	206291_at
CREB3L4	226455_at	PLAU	205479_s_at
DHCR24	200862_at		211668_s_at
EAF2	1568672_at	PMEPA1	217875_s_at
	1568673_s_at		222449_at

	219551_at		222450_at
ELL2	214446_at	PPAP2A	209147_s_at
	226099_at		210946_at
	226982_at	PRKACB	202741_at
FGF8	208449_s_at		202742_s_at
FKBP5	204560_at		235780_at
	224840_at	PTPN1	202716_at
	224856_at		217686_at
GUCY1A3	221942_s_at	SGK1	201739_at
	227235_at	TACC2	1570025_at
	229530_at		1570546_a_at
	239580_at		202289_s_at
IGF1	209540_at		211382_s_at
	209541_at	TMPRSS2	1570433_at
	209542_x_at		205102_at
	211577_s_at		211689_s_at
KLK2	1555545_at		226553_at
	209854_s_at	UGT2B15	207392_x_at
	209855_s_at		216687_x_at
	210339_s_at		

1B. AR target gene literature evidence and associated references

#	Gene Name	AR binds to regulatory region, **Experiment type [References]**	ARE motif in regulatory region, **Motif type[Reference]**	mRNA transcription **Experiment type [References]**	**Other**
1	KLK3	AR ChIP/PCR [1]–[4]<br>AR ChIP/seq [5], [6]$^{ARmo/hi}$, [2]<br>literature [7]<br>AR database[8]	confirmed experimentally, upstr. TSS:<br>- AGAACAgcaAGTGCT[7], [9]^{lit}<br>- AGGACAgtaAGCAAG[7]^{lit}<br>- GGAACAtatTGTATC[7],[9]^{lit}<br>- AGATCAaagAGATAA[7]^{lit}<br>- GGATCAgggAGTCTC[9]^{lit}<br>NI [3]^{Luc}, AR database[8]$^{4/2mut}$	PCR [5], [5]^{PT},[10], [11]$^{up\ ctr/nt\ AR\ siRNA}$<br>PCR [12]^{xen}<br>microarray [6]^{lit}, [1], [2], [7], [9], [10]<br>Northern blot [7]<br>RNA PolII ChIP/PCR [1] [3]<br>AR database[8]$^{19up/1down}$	Forkhead, GATA, and OCt motif[3]
2	KLK2	AR ChIP/PCR [1]<br>AR ChIP/CHIP [2]<br>AR ChIP/seq [5]<br>literature [7]<br>AR database[8]	confirmed experimentally, uptsr. TSS:<br>- GGAACAgcaAGTGCT [7] [9]^{lit}<br>- tggaGGAACAtatTGTATTtatt[13]$^{CAT/mut}$<br>AR database[8]$^{1/1mut}$	PCR [5], [5]^{PT}, [12]$^{xenogr.}$<br>microarray [1], [7], [9], [10]<br>RNA PolII ChIP/PCR [1]<br>AR database[8]^{12up}	
2	TMPRSS 2	AR ChIP/PCR[1], [3], [4]<br>AR ChIP/CHIP [3]<br>AR ChIP/seq [2]<br>AR database[8]^{3}	confirmed experimentally, upstr. TSS:<br>ACAACA 8 TGTCCT [3]$^{Luc/mut}$<br>putative: up. TSS: AGCAGAttcTGGTCT [7]	PCR [5], [5]^{PT}, [3]<br>microarray [1], [2]$^{<5h}$, [3], [7]<br>Northern blot [7]<br>RNA PolII ChIP/PCR [1], [3]<br>AR database[8]$^{10up/1down}$	GATA and OCt motif[3]
2	FKBP5	AR ChIP/PCR[4]<br>AR ChIP/PCR [14]^{mice}<br>AR ChIP/CHIP[15]^{HPr1AR}<br>AR ChIP/seq [5] [2] [6]$^{ARmo/hi}$<br>AR database[8]	confirmed experimentally, downtsr. TSS:<br>AGAACAgggTGTTCT [14]$^{Luc/mut}$	PCR [5],[10], [15]^{HPr1AR}<br>microarray [6]^{lit}, [1], [2]$^{<5h}$,[7],<br>microarray [9]$^{up\ 10nM/down\ 1nM}$,<br>microarray [10]^{PT}, [15]^{HPr1AR}<br>RNA PolII ChIP/PCR [2]<br>immunohistochemistry [10]^{PT}	

#	Gene Name	AR binds to regulatory region, **Experiment type [References]**	ARE motif in regulatory region, **Motif type[Reference]**	mRNA transcription **Experiment type [References]**	**Other**
				AR database[8][14up]	
5	GUCY1A<br>3	AR ChIP/PCR [16]<br>AR ChIP/seq [6][ARmo/hi], [2]<br>AR database[8]	confirmed experimentally: downtsr. TSS:<br>...GTACAAATCTCCT...[16][Luc]	PCR/western blot[16]<br>immunohistochemistry [16][PT]<br>microarray [6][lit], [2], [7], [9], [10]<br>AR database[8][5up]	expression increases with PC stage [16][PT]
6	ABCC4	AR ChIP/PCR [17]<br>AR ChIP/seq [6][ARmo], [2]	putative: AGACCAgccTGAGCA [18]	PCR [17], [19];<br>microarray [6][lit], [1], [2], [7]<br>RNA PolII ChIP/PCR [2]<br>AR database[8][5up]<br>western blot [19]	
7	NDRG1	AR ChIP/CHIP [15][HPr1AR]<br>AR ChIP/seq [5], [6][ARhi], [2]<br>AR database[8]	putative, uptsr. TSS:<br>- TGATTAaacTGTTCT [7]<br>- AATACAcccTGTTCC [9]<br>- AATAGAttgTGTATT[9]	PCR [5], [1], [9], [15][HPr1AR]<br>microarray [6][lit], [2], [7], [9], [10]<br>Northern blot [9]<br>RNA PolII ChIP/PCR [2]<br>AR database[8][10up]	
8	IGF1	AR ChIP/PCR [4], [20]	confirmed experimentally, uptsr. TSS:<br>ATATCTTATTCCTCTTTG,<br>GGCACATAGTAGAGCTCA,<br>or half sites, [8][lit], [20] [Luc/EMSA]	PCR [20]<br>microarray [1][3h down/6-48h up]<br>AR database[8][2up]	
9	CDKN1A	AR ChIP/PCR [4]<br>literature [7]<br>AR database[8]	confirmed experimentally, uptsr. TSS:<br>AGCACGcgaGGTTCC [21][EMSA/mut],<br>[7][lit], [22][Luc*], [8][lit]	Northern/Western blot [21]<br>AR database[8][4up/2down]	SP1 motif binds [22]
10	SGK1	AR ChIP/CHIP [15][HPr1AR]<br>AR ChIP/seq [6][ARmo/hi], [2]<br>AR database[8][5]	putative,<br>uptsr. TSS: GGCTATcccTGTTCT [8][lit]	PCR/microarray [15][HPr1AR]<br>microarray [6][lit], [1],[2][<5h], [7]<br>RNA PolII ChIP/PCR [2];<br>AR database[8][5up]	
11	DHCR24	AR ChIP/CHIP [15][HPr1AR]	putative,	Microarray [1], [2], [7], [10]	

#	Gene Name	AR binds to regulatory region, **Experiment type [References]**	ARE motif in regulatory region, **Motif type[Reference]**	mRNA transcription **Experiment type [References]**	**Other**
		AR ChIP/seq [2]<br>AR database[8]	uptsr. TSS: AGAACAtccTATTCC [7]	Northern blot [7]<br>AR database[8]^{5up}	
12	NKX3_1	AR ChIP/seq [2]<br>AR database[8]	putative,<br>uptsr. TSS: AGAACCattTGATGT [7]	microarray [1], [7], [9]<br>AR database[8]^{11up}	
13	APP	AR ChIP/PCR [23]<br>AR ChIP/CHIP [23]	confirmed experimentally,<br>- promoter:<br>AGAACAtgaAGCTAC [23]$^{Luc/mut}$<br>- intron 1:<br>GGTACTgacTGTATT [23]^{Luc}	PCR [23]$^{up>12 h}$<br>microarray [10]^{down}<br>PolII ChIP/PCR[23]<br>AcH3 ChIP/PCR[23]<br>AcH3 ChIP/CHIP[23]	Cancer marker [23]
14	AR	literature [7]	confirmed experimentally:<br>-exon D: CTTTCTgaaTGTCCT [7]^{lit}<br>-exon E: AGTACTcctGGATGG [7]^{lit}<br>AR database[8]^{3lit}	PCR [11]$^{up\ ctr/nt\ AR\ siRNA}$<br>AR database[8]$^{5up/8down}$<br>microarray[1]$^{3h\ up/6\text{-}48h\ down}$, [2]^{down}, [7]	
15	PMEPA1	AR ChIP/CHIP [15]^{HPr1AR}, [6]$^{ARmo/hi}$<br>AR database [8]^{2}	putative,<br>uptsr. TSS: TGAAGAatgTGTTCT [7]	PCR [15]^{HPr1AR}<br>microarray [6]^{lit}, [2]$^{<5h}$, [7],<br>AR database[8]$^{7up/2down}$	
16	LCP1	AR ChIP/seq [6]$^{ARmo/hi}$, [2]<br>AR database [8]		microarray [6]^{lit}, [2], [9]<br>RNA PolII ChIP/PCR [2]<br>AR database[8]^{4up}	
17	LRIG1	AR ChIP/seq [2], [6]$^{ARmo/hi}$		microarray [6]^{lit}, [1], [2], [11]^{ctr}<br>AR database[8]^{2up}	
18	PTPN1	AR ChIP/seq [2]		microarray [1]^{down}, [2]^{down}, [10]^{down}<br>RNA PolII ChIP/PCR [2]	
20	ELL2	AR ChIP/CHIP [15]^{HPr1AR}		PCR [15]^{HPr1AR}, [12]^{xen}, [14]^{mice}<br>microarray [1], [2]$^{<5h}$, [7], [9], [10]<br>AR database [8]^{6up}	
19	UGT2B15	AR database[8]		microarray [10]^{down}<br>AR database [8]$^{1up/9down}$	

#	**Gene Name**	AR binds to regulatory region, **Experiment type [References]**	ARE motif in regulatory region, **Motif type[Reference]**	mRNA transcription **Experiment type [References]**	**Other**
21	PPAP2A	AR ChIP/seq [6]$^{ARmo/hi}$		microarray [6]^{lit}, [1]$^{3h\ down/6\text{-}48h\ up}$, [2], [7]<br>AR database [8]^{3up}	
22	TACC2	AR ChIP/seq [5], [6]^{ARhi}, [2]		microarray [6]^{lit}, [2]<br>RNA PolII ChIP/PCR [2]	
23	EAF2	AR ChIP/seq [2]		microarray [1], [2]$^{<5h}$<br>RNA PolII ChIP/seq [2]	
24	FGF8	AR database [8]	putative: gcaGGGCCTggcTGTGCTgct [8]	AR database [8]^{2up}	
25	PLAU	AR CAT assay [24]<br>AR database [8]		PCR/microarray [15]$^{HPr1AR,\ down}$<br>northern blot [24]$^{PC3\text{-}AR\ down,\ CHX}$<br>western blot [24] $^{PC3\text{-}AR\ down}$<br>AR database [8]^{5down}	
26	CREB3L4			microarray [1]$^{3\text{-}6h\ down/12\text{-}48h\ up}$<br>Northern blot [25]$^{up,chx\ down,\ PT}$<br>Immunostaining [25]$^{PT\ up}$, [26]<br>AR database [8]^{1up}	Indirect gene [25]
27	PRKACB			PCR/ microarray [27],<br>immunoblot [27], [27]^{PT}<br>AR database [8]^{1up}	
28	NTS			Northern blot [28]^{down}<br>microarray [2]^{down}	
NA	DRG1				

(PT): measured in primary tumor; (up): up regulated- only indicated if needed; (down): down regulated; (#): number of references cited;
(#h) exposure time; (#nM) concentration; (nt AR siRNA): not differentially expressed when AR is silenced;
(ctr): in control siRNA experiment; (CHX): in cycloheximide
(ARmo): LNCaP cell line moderately overexpressing AR; (AR /hi): LNCaP cell line highly one\overexpressing AR; (PC3-AR) PC3 cell line expressing AR;
(lit): from analysis of public microarray data;

(HPr1AR): normal PC cell line overexpressing AR; (xen) measured in xenograft; (mice) measured in mice;
TSS: Transcription site start

2A. TGFβ pathway target gene probeset list

Gene	Probeset	Gene	Probeset
ANGPTL4	223333_s_at	PDGFB	204200_s_at
	221009_s_at		216061_x_at
CDC42EP3	209286_at		217112_at
	209288_s_at		217430_x_at
	225685_at	PTHLH	210355_at
	209287_s_at		206300_s_at
CDKN1A	202284_s_at		1556773_at
	1555186_at		211756_at
CDKN2B	236313_at	SERPINE1	202627_s_at
	207530_s_at		1568765_at
CTGF	209101_at		202628_s_at
GADD45A	203725_at	SGK1	201739_at
GADD45B	207574_s_at	SKIL	206675_s_at
	209305_s_at		225227_at
	209304_x_at		215889_at
HMGA2	208025_s_at	SMAD4	202526_at
	1567224_at		202527_s_at
	1568287_at		1565703_at
	1558683_a_at		235725_at
	1561633_at	SMAD5	225223_at
	1559891_at		235451_at
	1558682_at		225219_at
ID1	208937_s_at		205187_at
IL11	206924_at		205188_s_at
	206926_s_at	SMAD6	207069_s_at
INPP5D	203331_s_at		209886_s_at
	1568943_at	SMAD7	204790_at
	203332_s_at	SNAI1	219480_at
JUNB	201473_at	SNAI2	213139_at
MMP2	1566678_at		
	201069_at	VEGFA	210513_s_at
MMP9	203936_s_at		210512_s_at
NKX2-5	206578_at		212171_x_at
OVOL1	206604_at		211527_x_at
	229396_at		

2B. TGFbeta pathway target gene selection, literature evidence and associated references

RE: Response element (enhancer element/binding motif)

PR: promoter reporter (e.g. luciferase) experiment

ChIP: Chromatin Immuno Precipitation

EMSA: Electrophoretic Mobility Shift Assay

DE: differential expression between samples with active and inactive pathway

Score: cumulative score

Target genes	Reference	RE	PR	ChIP	DE	Score
ANGPTL4	[29],[30],	1			1	2
CDC42EP3	[31]	1			1	2
CDKN1A	[31],[32],[33]	1			1	2
CDKN2B	[31];[32],[34]	1				1
CTGF	[31],[35], [36],[37],[29]	1			1	2
GADD45A	[31]	1			1	2
HMGA2	[29],[38],[39]	1	1	1	1	3
ID1	[40],[41],[29]	1	1		1	2
IL11	[35],[42]				1	1
INPP5D	[43]				1	1
JUNB	[44],[45],[46],[39]	1	1		1	3
MMP2	[47],[48]				1	1
MMP9	[47],[48]				1	1
NKX2-5	[49]	1			1	2
OVOL1	[31]	1			1	2
PDGFB	[31],[29]	1			1	2
PTHLH	[50],[35],[29]	1			1	2
SERPINE1	[37],[36],[51]	1			1	2
SGK1	[31]	1			1	2
SKIL	[52]				1	1
SMAD4	[33]	1			1	2
SMAD5	[53]	1				1
SMAD7	[54],[33],[51]	1			1	2
SNAI1	[39],[29]	1			1	2
TIMP1	[48]				1	1
VEGFA	[29],[29],[55],[35]	1	1		1	4

Additional general references: [56];[57];[58];[59],[60]

3A. Hedgehog pathway target gene probeset list

Gene	Probeset	Gene	Probeset
BCL2	203684_s_at	HHIP	1556037_s_at
	203685_at		223775_at
	207004_at		237466_s_at
	207005_s_at	IGFBP6	203851_at
CCND1	208711_s_at	IL1R2	205403_at
	208712_at		211372_s_at
	214019_at	JAG2	209784_s_at
CCND2	200951_s_at		32137_at
	200952_s_at	JUP	201015_s_at
	200953_s_at		
	231259_s_at	MYCN	209756_s_at
CFLAR	208485_x_at		209757_s_at
	209508_x_at		211377_x_at
	209939_x_at		234376_at
	210563_x_at	MYLK	1563466_at
	210564_x_at		1568770_at
	211316_x_at		202555_s_at
	211317_s_at		224823_at
	211862_x_at	NKX2-2	206915_at
	214486_x_at	NKX2-8	207451_at
	214618_at	PITRM1	205273_s_at
	235427_at	PTCH1	1555520_at
	239629_at		208522_s_at
	224261_at		209815_at
CTSL	202087_s_at		209816_at
FOXA2	210103_s_at	PTCH2	221292_at
	40284_at	RAB34	1555630_a_at
FOXF1	205935_at		224710_at
FOXL1	216572_at	S100A7	205916_at
	243409_at	S100A9	203535_at
FOXM1	202580_x_at	SPP1	209875_s_at
FST	204948_s_at	TCEA2	203919_at
	207345_at		238173_at
	226847_at		241428_x_at
FYN	1559101_at	TOM1	202807_s_at
	210105_s_at	TSC22D1	215111_s_at
	212486_s_at		243133_at
	216033_s_at		239123_at
GLI1	206646_at		

GLI3	1569342_at
	205201_at
	227376_at
H19	224646_x_at
	224997_x_at

3B. Hedgehog pathway target gene selection, literature evidence and references

RE: Response element (enhancer element/binding motif)

PR: promoter reporter (e.g. luciferase) experiment

ChIP: Chromatin Immuno Precipitation

EMSA: Electrophoretic Mobility Shift Assay

DE: differential expression between samples with active and inactive pathway

CHX: differential expression in the presence of cycloheximide

TF: transcription factor

Score: cumulative score

NAME	REFERENCES	PR	RE	EMSA	CHIP	DE	CHX	stable trans-fection TF	SCORE
GLI1	[61],[62],[63],[64],[65]		1	1	1	1			4
PTCH1	[61],[62],[63],[64],[65]		1		1	1			3
PTCH2	[61], [62]		1		1	1			3
HHIP	[62]		1		1				2
SPP1	[61]		1	1		1		1	3
TSC22D1	[61]					1		1	2
H19	[61]					1		1	2
IGFBP6	[61]		1	1		1		1	4
TOM1	[61]					1		1	2
JUP	[61],[63]		1	1		1			3
nkx2-2	[62],[63]		1		1				2
nkx2-8	[62]		1		1				2
FoxA2	[62]				1				1

Rab34	[62]		1		1				2
GLI3	[62]				1				1
FST	[66]	1	1	1		1			4
BCL2	[67],[68]	1	1	1		1			4
CTSL	[67]					1			1
TCEA2	[67]					1			1
MYLK	[67]					1			1
FYN	[67]					1			1
PITRM1	[67]					1			1
CFLAR	[69],[70]	1	1	1		1			4
IL1R2	[67]	1	1						2
S100A7	[67]	1	1						2
S100A9	[67]	1	1						2
CCND1	[67]	1	1						2
JAG2	[67]	1	1						2
FOXM1	[71]					1			1
Foxf1	[72]	1	1	1	1	1			5
FoxL1	[70]		1	1	1	1			4
CCND2	[63],[70]		1		1				2
MYCN	[73],[70]		1			1	1		3

4A. NFkB pathway target gene selection, Affymetrix probeset selection, and associated references

RE: Response element (enhancer element/binding motif)

PR: promoter reporter (e.g. luciferase) experiment

ChIP: Chromatin Immuno Precipitation

EMSA: Electrophoretic Mobility Shift Assay

DE: differential expression between samples with active and inactive pathway

CHX differential expression in the presence of cycloheximide

Score: cumulative score

**Gene**	**Probeset**	RE	PR	ChIP	EMSA	DE	CHX	Score	REFERENCES
PTGS2	1554997_a_at	1	1	1		1		4	[74],[75],[76],[77],[78],[79],[80]
	204748_at								
IL8	202859_x_at	1	1	1		1		2	[80],[81],[82],[78],[83]
	211506_s_at								
NFKBIE	203927_at	1	1	1		1	1	5	[83],[84] [82]
IL6	205207_at	1		1		1		3	[76],[85],[86],[84]
MMP9	203936_s_at	1		1				2	[78]
TNF	207113_s_at	1	1		1	1		4	[76],[78],[82], [81]
ICAM1	202637_s_at	1	1	1		1		4	[80], [86],[85], [78],[77]

	202638_s_at								
	215485_s_at								
CCL2	216598_s_at	1			1	1		3	[82],[86],[77],[87]
CCL5	1405_i_at	1	1		1	1		4	[78],[84],[81],[88]
	1555759_a_at								
	204655_at								
NFKB2	207535_s_at	1		1		1		3	[88],[76],[78],[89],[84],<br>[84],[82]
	209636_at								
	211524_at								
TNIP1	207196_s_at	1		1	1	1	1	5	[83],[88],[76],[89]
VCAM1	203868_s_at	1	1			1		3	[86],[77],[75]
SELE	206211_at	1				1		2	[77],[85],[82]
CCL22	207861_at	1		1				2	[81],[88]
BCL2L1	206665_s_at				1	1		2	[88],[75],[79]
	212312_at								
	215037_s_at								
	231228_at								

NFKBIA	201502_s_at	1				1		2	[76],[84],[82],[81]
	231699_at								
IRF1	202531_at	1		1		1		3	[88],[76],[78]
	238725_at								
CCL20	205476_at	1				1		2	[76],[86],[82]
CX3CL1	203687_at	1				1		2	[88],[77],[86]
	823_at								
CXCL2	1569203_at	1		1	1	1		4	[77],[76],[75]
	209774_x_at								
	230101_at								
CXCL3	207850_at	1		1	1	1		4	[83],[76],[78],[86]
	205114_s_at								
CXCL1	204470_at	1		1		1		3	[75],[84],[86],[78],[76]
IL1B	205067_at	1	1		1	1		4	[78],[82],[87]
	39402_at								
CCL4	204103_at	1		1		1		3	[78],[87]
BIRC3	210538_s_at	1			1	1		3	[88],[89],[79],[86],[82]
	230499_at								

TNFAIP2	202509_s_at	1			1	1	1	4	[81]
	202510_s_at								
STAT5A	203010_at	1		1		1		3	[88],[78]
TRAF1	205599_at	1		1		1		3	[88],[76],[77],[78],[79]
	235116_at								
CCL3	205114_s_at	1		1		1		3	[88],[76]

Reference list for selected AR, TGFbeta, HH, and NFκB pathway target genes

[1] J. C. Zhao *e.a.*, “Cooperation between Polycomb and androgen receptor during oncogenic transformation”, *Genome Res.*, vol. 22, nr. 2, pp. 322–331, feb. 2012.

[2] C. E. Massie *e.a.*, “The androgen receptor fuels prostate cancer by regulating central metabolism and biosynthesis: AR coordinates anabolic program in prostate cancer”, *EMBO J.*, vol. 30, nr. 13, pp. 2719–2733, jul. 2011.

[3] Q. Wang *e.a.*, “A Hierarchical Network of Transcription Factors Governs Androgen Receptor-Dependent Prostate Cancer Growth”, *Mol. Cell*, vol. 27, nr. 3, pp. 380–392, aug. 2007.

[4] “ChIP analysis of androgen regulation of transcriptional activity”, *Previews*. [Online]. Beschikbaar op: http://www.piercenet.com/previews/2013-articles/chip-analysis-androgen-regulation-transcription/. [Geraadpleegd: 09-apr-2014].

[5] J. Yu *e.a.*, “An Integrated Network of Androgen Receptor, Polycomb, and TMPRSS2-ERG Gene Fusions in Prostate Cancer Progression”, *Cancer Cell*, vol. 17, nr. 5, pp. 443–454, mei 2010.

[6] A. Urbanucci *e.a.*, “Overexpression of androgen receptor enhances the binding of the receptor to the chromatin in prostate cancer”, *Oncogene*, vol. 31, nr. 17, pp. 2153–2163, apr. 2012.

[7] P. S. Nelson *e.a.*, “The program of androgen-responsive genes in neoplastic prostate epithelium”, *Proc. Natl. Acad. Sci. U. S. A.*, vol. 99, nr. 18, pp. 11890–11895, sep. 2002.

[8] M. Jiang *e.a.*, “Androgen-responsive gene database: integrated knowledge on androgen-responsive genes”, *Mol. Endocrinol. Baltim. Md*, vol. 23, nr. 11, pp. 1927–1933, nov. 2009.

[9] T. Segawa *e.a.*, “Androgen-induced expression of endoplasmic reticulum (ER) stress response genes in prostate cancer cells”, *Oncogene*, vol. 21, nr. 57, pp. 8749–8758, dec. 2002.

[10] A. M. Velasco *e.a.*, “Identification and validation of novel androgen-regulated genes in prostate cancer”, *Endocrinology*, vol. 145, nr. 8, pp. 3913–3924, aug. 2004.

[11] H. V. Heemers *e.a.*, “Identification of a Clinically Relevant Androgen-Dependent Gene Signature in Prostate Cancer”, *Cancer Res.*, vol. 71, nr. 5, pp. 1978–1988, mrt. 2011.

[12] G. Wang, S. J. M. Jones, M. A. Marra, en M. D. Sadar, “Identification of genes targeted by the androgen and PKA signaling pathways in prostate cancer cells”, *Oncogene*, vol. 25, nr. 55, pp. 7311–7323, jun. 2006.

[13] S. H. Mitchell, P. E. Murtha, S. Zhang, W. Zhu, en C. Y. F. Young, “An androgen response element mediates LNCaP cell dependent androgen induction of the hK2 gene”, *Mol. Cell. Endocrinol.*, vol. 168, nr. 1–2, pp. 89–99, okt. 2000.

[14] J. A. Magee, L. Chang, G. D. Stormo, en J. Milbrandt, “Direct, androgen receptor-mediated regulation of the FKBP5 gene via a distal enhancer element”, *Endocrinology*, vol. 147, nr. 1, pp. 590–598, jan. 2006.

[15] E. C. Bolton, A. Y. So, C. Chaivorapol, C. M. Haqq, H. Li, en K. R. Yamamoto, “Cell- and gene-specific regulation of primary target genes by the androgen receptor”, *Genes Dev.*, vol. 21, nr. 16, pp. 2005–2017, aug. 2007.

[16] C. Cai *e.a.*, “Androgen regulation of soluble guanylyl cyclasealpha1 mediates prostate cancer cell proliferation”, *Oncogene*, vol. 26, nr. 11, pp. 1606–1615, mrt. 2007.

[17] D. Wu *e.a.*, “Three-tiered role of the pioneer factor GATA2 in promoting androgen-dependent gene expression in prostate cancer”, *Nucleic Acids Res.*, vol. 42, nr. 6, pp. 3607–3622, apr. 2014.

[18] S. Denayer, C. Helsen, L. Thorrez, A. Haelens, en F. Claessens, “The rules of DNA recognition by the androgen receptor”, *Mol. Endocrinol. Baltim. Md*, vol. 24, nr. 5, pp. 898–913, mei 2010.

[19] C. Cai, J. Omwancha, C.-L. Hsieh, en L. Shemshedini, “Androgen induces expression of the multidrug resistance protein gene MRP4 in prostate cancer cells”, *Prostate Cancer Prostatic Dis.*, vol. 10, nr. 1, pp. 39–45, sep. 2006.

[20] Y. Wu *e.a.*, “Identification of androgen response elements in the insulin-like growth factor I upstream promoter”, *Endocrinology*, vol. 148, nr. 6, pp. 2984–2993, jun. 2007.

[21] S. Lu, M. Liu, D. E. Epner, S. Y. Tsai, en M. J. Tsai, “Androgen regulation of the cyclin-dependent kinase inhibitor p21 gene through an androgen response element in the proximal promoter”, *Mol. Endocrinol. Baltim. Md*, vol. 13, nr. 3, pp. 376–384, mrt. 1999.

[22] S. Lu, G. Jenster, en D. E. Epner, “Androgen induction of cyclin-dependent kinase inhibitor p21 gene: role of androgen receptor and transcription factor Sp1 complex”, *Mol. Endocrinol. Baltim. Md*, vol. 14, nr. 5, pp. 753–760, mei 2000.

[23] K. Takayama *e.a.*, “Amyloid Precursor Protein Is a Primary Androgen Target Gene That Promotes Prostate Cancer Growth”, *Cancer Res.*, vol. 69, nr. 1, pp. 137–142, jan. 2009.

[24] C. P. Evans, E. C. Stapp, M. A. Dall’Era, J. Juarez, en J. C. Yang, “Regulation of u-PA gene expression in human prostate cancer”, *Int. J. Cancer J. Int. Cancer*, vol. 94, nr. 3, pp. 390–395, nov. 2001.

[25] H. Qi *e.a.*, “AIbZIP, a novel bZIP gene located on chromosome 1q21.3 that is highly expressed in prostate tumors and of which the expression is up-regulated by androgens in LNCaP human prostate cancer cells”, *Cancer Res.*, vol. 62, nr. 3, pp. 721–733, feb. 2002.

[26] M.-H. Levesque, M. El-Alfy, L. Berger, F. Labrie, en C. Labrie, “Evaluation of AIbZIP and Cdc47 as markers for human prostatic diseases”, *Urology*, vol. 69, nr. 1, pp. 196–201, jan. 2007.

[27] A.-K. Kvissel, H. Ramberg, T. Eide, A. Svindland, B. S. Skålhegg, en K. A. Taskén, “Androgen dependent regulation of protein kinase A subunits in prostate cancer cells”, *Cell. Signal.*, vol. 19, nr. 2, pp. 401–409, feb. 2007.

[28] I. Sehgal, S. Powers, B. Huntley, G. Powis, M. Pittelkow, en N. J. Maihle, “Neurotensin is an autocrine trophic factor stimulated by androgen withdrawal in human prostate cancer”, *Proc. Natl. Acad. Sci.*, vol. 91, nr. 11, pp. 4673–4677, mei 1994.

[29] D. Padua en J. Massagué, “Roles of TGFbeta in metastasis”, *Cell Res.*, vol. 19, nr. 1, pp. 89–102, jan. 2009.

[30] D. Padua *e.a.*, “TGFbeta primes breast tumors for lung metastasis seeding through angiopoietin-like 4”, *Cell*, vol. 133, nr. 1, pp. 66–77, apr. 2008.

[31] R. R. Gomis *e.a.*, “A FoxO-Smad synexpression group in human keratinocytes”, *Proc. Natl. Acad. Sci. U. S. A.*, vol. 103, nr. 34, pp. 12747–12752, aug. 2006.

[32] C. Hesling *e.a.*, “Antagonistic regulation of EMT by TIF1γ and Smad4 in mammary epithelial cells”, *EMBO Rep.*, vol. 12, nr. 7, pp. 665–672, jul. 2011.

[33] J. Seoane, H.-V. Le, L. Shen, S. A. Anderson, en J. Massagué, “Integration of Smad and forkhead pathways in the control of neuroepithelial and glioblastoma cell proliferation”, *Cell*, vol. 117, nr. 2, pp. 211–223, apr. 2004.

[34] M. B. Buck en C. Knabbe, “TGF-beta signaling in breast cancer”, *Ann. N. Y. Acad. Sci.*, vol. 1089, pp. 119–126, nov. 2006.

[35] M. Petersen *e.a.*, “Smad2 and Smad3 have opposing roles in breast cancer bone metastasis by differentially affecting tumor angiogenesis”, *Oncogene*, vol. 29, nr. 9, pp. 1351–1361, mrt. 2010.

[36] K. S. Hoek *e.a.*, “Metastatic potential of melanomas defined by specific gene expression profiles with no BRAF signature”, *Pigment Cell Res.*, vol. 19, nr. 4, pp. 290–302, aug. 2006.

[37] R. Samarakoon *e.a.*, “Induction of renal fibrotic genes by TGF-β1 requires EGFR activation, p53 and reactive oxygen species”, *Cell. Signal.*, vol. 25, nr. 11, pp. 2198–2209, nov. 2013.

[38] S. Thuault, U. Valcourt, M. Petersen, G. Manfioletti, C.-H. Heldin, en A. Moustakas, “Transforming growth factor-beta employs HMGA2 to elicit epithelial-mesenchymal transition”, *J. Cell Biol.*, vol. 174, nr. 2, pp. 175–183, jul. 2006.

[39] E. Kubo, S. Shibata, T. Shibata, E. Kiyokawa, H. Sasaki, en D. P. Singh, “FGF2 antagonizes aberrant TGFβ regulation of tropomyosin: role for posterior capsule opacity”, *J. Cell. Mol. Med.*, vol. 21, nr. 5, pp. 916–928, 2017.

[40] T. Katagiri en S. Tsukamoto, “The unique activity of bone morphogenetic proteins in bone: a critical role of the Smad signaling pathway”, *Biol. Chem.*, vol. 394, nr. 6, pp. 703–714, jun. 2013.

[41] E. Wiercinska *e.a.*, “Id1 is a critical mediator in TGF-beta-induced transdifferentiation of rat hepatic stellate cells”, *Hepatol. Baltim. Md*, vol. 43, nr. 5, pp. 1032–1041, mei 2006.

[42] A. Calon *e.a.*, “Dependency of colorectal cancer on a TGF-β-driven program in stromal cells for metastasis initiation”, *Cancer Cell*, vol. 22, nr. 5, pp. 571–584, nov. 2012.

[43] F. Li, L. Li, J. Hao, S. Liu, en H. Duan, “Src Homology 2 Domain-Containing Inositol 5’-Phosphatase Ameliorates High Glucose-Induced Extracellular Matrix Deposition via the Phosphatidylinositol 3-Kinase/Protein Kinase B Pathway in Renal Tubular Epithelial Cells”, *J. Cell. Biochem.*, vol. 118, nr. 8, pp. 2271–2284, 2017.

[44] L. J. Jonk, S. Itoh, C. H. Heldin, P. ten Dijke, en W. Kruijer, “Identification and functional characterization of a Smad binding element (SBE) in the JunB promoter that acts as a transforming growth factor-beta, activin, and bone morphogenetic protein-inducible enhancer”, *J. Biol. Chem.*, vol. 273, nr. 33, pp. 21145–21152, aug. 1998.

[45] K.-S. Lee, S.-H. Hong, en S.-C. Bae, “Both the Smad and p38 MAPK pathways play a crucial role in Runx2 expression following induction by transforming growth factor-beta and bone morphogenetic protein”, *Oncogene*, vol. 21, nr. 47, pp. 7156–7163, okt. 2002.

[46] F. Verrecchia, C. Tacheau, M. Schorpp-Kistner, P. Angel, en A. Mauviel, “Induction of the AP-1 members c-Jun and JunB by TGF-beta/Smad suppresses early Smad-driven gene activation”, *Oncogene*, vol. 20, nr. 18, pp. 2205–2211, apr. 2001.

[47] M. Pickup, S. Novitskiy, en H. L. Moses, “The roles of TGFβ in the tumour microenvironment”, *Nat. Rev. Cancer*, vol. 13, nr. 11, pp. 788–799, nov. 2013.

[48] H.-J. Kwak *e.a.*, “Transforming growth factor-beta1 induces tissue inhibitor of metalloproteinase-1 expression via activation of extracellular signal-regulated kinase and Sp1 in human fibrosarcoma cells”, *Mol. Cancer Res. MCR*, vol. 4, nr. 3, pp. 209–220, mrt. 2006.

[49] C. O. Brown, X. Chi, E. Garcia-Gras, M. Shirai, X.-H. Feng, en R. J. Schwartz, “The cardiac determination factor, Nkx2-5, is activated by mutual cofactors GATA-4 and Smad1/4 via a novel upstream enhancer”, *J. Biol. Chem.*, vol. 279, nr. 11, pp. 10659–10669, mrt. 2004.

[50] Y. Y. Sheen, M.-J. Kim, S.-A. Park, S.-Y. Park, en J.-S. Nam, “Targeting the Transforming Growth Factor-β Signaling in Cancer Therapy”, *Biomol. Ther.*, vol. 21, nr. 5, pp. 323–331, sep. 2013.

[51] J. Massagué en Q. Xi, “TGF-β control of stem cell differentiation genes”, *FEBS Lett.*, vol. 586, nr. 14, pp. 1953–1958, jul. 2012.

[52] J. Massagué, J. Seoane, en D. Wotton, “Smad transcription factors”, *Genes Dev.*, vol. 19, nr. 23, pp. 2783–2810, dec. 2005.

[53] A. Moustakas, S. Souchelnytskyi, en C. H. Heldin, “Smad regulation in TGF-beta signal transduction”, *J. Cell Sci.*, vol. 114, nr. Pt 24, pp. 4359–4369, dec. 2001.

[54] L. Ciuclan *e.a.*, “TGF-beta enhances alcohol dependent hepatocyte damage via down-regulation of alcohol dehydrogenase I”, *J. Hepatol.*, vol. 52, nr. 3, pp. 407–416, mrt. 2010.

[55] T. Sánchez-Elsner, L. M. Botella, B. Velasco, A. Corbí, L. Attisano, en C. Bernabéu, “Synergistic cooperation between hypoxia and transforming growth factor-beta pathways on human vascular endothelial growth factor gene expression”, *J. Biol. Chem.*, vol. 276, nr. 42, pp. 38527–38535, okt. 2001.

[56] J. E. Chipuk *e.a.*, “The androgen receptor represses transforming growth factor-beta signaling through interaction with Smad3”, *J. Biol. Chem.*, vol. 277, nr. 2, pp. 1240–1248, jan. 2002.

[57] B. I. Dalal, P. A. Keown, en A. H. Greenberg, “Immunocytochemical localization of secreted transforming growth factor-beta 1 to the advancing edges of primary tumors and to lymph node metastases of human mammary carcinoma”, *Am. J. Pathol.*, vol. 143, nr. 2, pp. 381–389, aug. 1993.

[58] Y. Kang *e.a.*, “A multigenic program mediating breast cancer metastasis to bone”, *Cancer Cell*, vol. 3, nr. 6, pp. 537–549, jun. 2003.

[59] L. Mishra, R. Derynck, en B. Mishra, “Transforming growth factor-beta signaling in stem cells and cancer”, *Science*, vol. 310, nr. 5745, pp. 68–71, okt. 2005.

[60] P. M. Siegel en J. Massagué, “Cytostatic and apoptotic actions of TGF-beta in homeostasis and cancer”, *Nat. Rev. Cancer*, vol. 3, nr. 11, pp. 807–821, nov. 2003.

[61] J. W. Yoon *e.a.*, “Gene Expression Profiling Leads to Identification of GLI1-binding Elements in Target Genes and a Role for Multiple Downstream Pathways in GLI1-induced Cell Transformation”, *J. Biol. Chem.*, vol. 277, nr. 7, pp. 5548–5555, feb. 2002.

[62] S. A. Vokes, H. Ji, W. H. Wong, en A. P. McMahon, “A genome-scale analysis of the cis-regulatory circuitry underlying sonic hedgehog-mediated patterning of the mammalian limb”, *Genes Dev.*, vol. 22, nr. 19, pp. 2651–2663, okt. 2008.

[63] M. H. Shahi *e.a.*, “Regulation of sonic hedgehog-GLI1 downstream target genes PTCH1, Cyclin D2, Plakoglobin, PAX6 and NKX2.2 and their epigenetic status in medulloblastoma and astrocytoma”, *BMC Cancer*, vol. 10, p. 614, nov. 2010.

[64] F. Götschel *e.a.*, “Synergism between Hedgehog-GLI and EGFR signaling in Hedgehog-responsive human medulloblastoma cells induces downregulation of canonical Hedgehog-target genes and stabilized expression of GLI1”, *PloS One*, vol. 8, nr. 6, p. e65403, 2013.

[65] A. Shaw, J. Gipp, en W. Bushman, “The Sonic Hedgehog pathway stimulates prostate tumor growth by paracrine signaling and recapitulates embryonic gene expression in tumor myofibroblasts”, *Oncogene*, vol. 28, nr. 50, pp. 4480–4490, dec. 2009.

[66] T. Eichberger *e.a.*, “GLI2-specific transcriptional activation of the bone morphogenetic protein/activin antagonist follistatin in human epidermal cells”, *J. Biol. Chem.*, vol. 283, nr. 18, pp. 12426–12437, mei 2008.

[67] M. Kasper *e.a.*, “Selective modulation of Hedgehog/GLI target gene expression by epidermal growth factor signaling in human keratinocytes”, *Mol. Cell. Biol.*, vol. 26, nr. 16, pp. 6283–6298, aug. 2006.

[68] G. Regl *e.a.*, “Activation of the BCL2 promoter in response to Hedgehog/GLI signal transduction is predominantly mediated by GLI2”, *Cancer Res.*, vol. 64, nr. 21, pp. 7724–7731, nov. 2004.

[69] E. Kump, J. Ji, M. Wernli, P. Häusermann, en P. Erb, “Gli2 upregulates cFlip and renders basal cell carcinoma cells resistant to death ligand-mediated apoptosis”, *Oncogene*, vol. 27, nr. 27, pp. 3856–3864, jun. 2008.

[70] Y. Katoh en M. Katoh, “Hedgehog target genes: mechanisms of carcinogenesis induced by aberrant hedgehog signaling activation”, *Curr. Mol. Med.*, vol. 9, nr. 7, pp. 873–886, sep. 2009.

[71] D. Wang *e.a.*, “Aberrant activation of hedgehog signaling promotes cell proliferation via the transcriptional activation of forkhead Box M1 in colorectal cancer cells”, *J. Exp. Clin. Cancer Res. CR*, vol. 36, nr. 1, p. 23, 02 2017.

[72] T. Bohnenpoll *e.a.*, “A SHH-FOXF1-BMP4 signaling axis regulating growth and differentiation of epithelial and mesenchymal tissues in ureter development”, *PLoS Genet.*, vol. 13, nr. 8, p. e1006951, aug. 2017.

[73] A. M. Kenney en D. H. Rowitch, “Sonic hedgehog promotes G(1) cyclin expression and sustained cell cycle progression in mammalian neuronal precursors”, *Mol. Cell. Biol.*, vol. 20, nr. 23, pp. 9055–9067, dec. 2000.

[74] A. Yoshimura, “Signal transduction of inflammatory cytokines and tumor development”, *Cancer Sci.*, vol. 97, nr. 6, pp. 439–447, jun. 2006.

[75] K. I. Amiri en A. Richmond, “Role of nuclear factor-kappa B in melanoma”, *Cancer Metastasis Rev.*, vol. 24, nr. 2, pp. 301–313, jun. 2005.

[76] B. Tian, D. E. Nowak, en A. R. Brasier, “A TNF-induced gene expression program under oscillatory NF-kappaB control”, *BMC Genomics*, vol. 6, p. 137, sep. 2005.

[77] T. Minami, A. Sugiyama, S.-Q. Wu, R. Abid, T. Kodama, en W. C. Aird, “Thrombin and phenotypic modulation of the endothelium”, *Arterioscler. Thromb. Vasc. Biol.*, vol. 24, nr. 1, pp. 41–53, jan. 2004.

[78] J. Schreiber, R. G. Jenner, H. L. Murray, G. K. Gerber, D. K. Gifford, en R. A. Young, “Coordinated binding of NF-kappaB family members in the response of human cells to lipopolysaccharide”, *Proc. Natl. Acad. Sci. U. S. A.*, vol. 103, nr. 15, pp. 5899–5904, apr. 2006.

[79] G. Sethi, K. S. Ahn, S. K. Sandur, X. Lin, M. M. Chaturvedi, en B. B. Aggarwal, “Indirubin enhances tumor necrosis factor-induced apoptosis through modulation of nuclear factor-kappa B signaling pathway”, *J. Biol. Chem.*, vol. 281, nr. 33, pp. 23425–23435, aug. 2006.

[80] R. Martone *e.a.*, “Distribution of NF-kappaB-binding sites across human chromosome 22”, *Proc. Natl. Acad. Sci. U. S. A.*, vol. 100, nr. 21, pp. 12247–12252, okt. 2003.

[81] S. Saccani, S. Pantano, en G. Natoli, “Modulation of NF-kappaB activity by exchange of dimers”, *Mol. Cell*, vol. 11, nr. 6, pp. 1563–1574, jun. 2003.

[82] M. R. Milward, I. L. C. Chapple, H. J. Wright, J. L. Millard, J. B. Matthews, en P. R. Cooper, “Differential activation of NF-kappaB and gene expression in oral epithelial cells by periodontal pathogens”, *Clin. Exp. Immunol.*, vol. 148, nr. 2, pp. 307–324, mei 2007.

[83] B. Tian, D. E. Nowak, M. Jamaluddin, S. Wang, en A. R. Brasier, “Identification of direct genomic targets downstream of the nuclear factor-kappaB transcription factor mediating tumor necrosis factor signaling”, *J. Biol. Chem.*, vol. 280, nr. 17, pp. 17435–17448, apr. 2005.

[84] H. J. Choi *e.a.*, “Genome-wide identification of palmitate-regulated immediate early genes and target genes in pancreatic beta-cells reveals a central role of NF-κB”, *Mol. Biol. Rep.*, vol. 39, nr. 6, pp. 6781–6789, jun. 2012.

[85] G. B. Fogel *e.a.*, “Discovery of sequence motifs related to coexpression of genes using evolutionary computation”, *Nucleic Acids Res.*, vol. 32, nr. 13, pp. 3826–3835, 2004.

[86] D. Viemann *e.a.*, “The contact allergen nickel triggers a unique inflammatory and proangiogenic gene expression pattern via activation of NF-kappaB and hypoxia-inducible factor-1alpha”, *J. Immunol. Baltim. Md 1950*, vol. 178, nr. 5, pp. 3198–3207, mrt. 2007.

[87] M. Delgado en D. Ganea, “Inhibition of endotoxin-induced macrophage chemokine production by vasoactive intestinal peptide and pituitary adenylate cyclase-activating polypeptide in vitro and in vivo”, *J. Immunol. Baltim. Md 1950*, vol. 167, nr. 2, pp. 966–975, jul. 2001.

[88] M. Hinz *e.a.*, “Nuclear factor kappaB-dependent gene expression profiling of Hodgkin’s disease tumor cells, pathogenetic significance, and link to constitutive signal transducer and activator of transcription 5a activity”, *J. Exp. Med.*, vol. 196, nr. 5, pp. 605–617, sep. 2002.

[89] R. Elkon, C. Linhart, Y. Halperin, Y. Shiloh, en R. Shamir, “Functional genomic delineation of TLR-induced transcriptional networks”, *BMC Genomics*, vol. 8, p. 394, okt. 2007.

Comparison between Gene Set Enrichment Analysis (GSEA) and Pathway analysis on two groups of samples in a dataset

GEO dataset GSE8671 contains Affymetrix HG-U133Plus2.0 data of 64 patient samples: 32 colon adenomas and 32 matched normal colon samples. The Wnt pathway is known to be the dominant signaling pathway that is active in colon adenoma and carcinoma (e.g. work of Hans Clevers, e.g. Bienz M, Clevers H, Cell. 2000 Oct 13;103(2):311-20; Radtke F, Clevers H, Science. 2005 Mar 25;307(5717):1904-9.)
Analysis of individual sample data with our pathway analysis approach, revealed the Wnt pathway as the most prominently active pathway, with the Wnt pathway active in colon adenoma samples and inactive in normal colon samples; the other dominant pathway was the PI3K pathway, which was found active in the majority of the colon adenoma samples, and not in the normal tissue samples (Verhaegh et al., Cancer Res. 2014 Jun 1;74(11):2936-45; and van Ooijen et all. Am J Pathol. 2018 Sep;188(9):1956-1972).
GSEA was performed on the two groups of samples (colon adenoma versus normal colon) resulting in the Wnt pathway showing up for the first time at rank 87, with a false-discovery rate corrected p-value of 0.038, while other Wnt-related pathways were not significantly upregulated (see Table A below). The PI3K pathway did not show up as a significantly upregulated (Table D). In contrast, many other pathways were listed in the top 10 up- or downregulated pathways without any clear biological relation to colon adenomas vs. normal colon (Tables B-C).

Results GSEA:

A. Ranks of upregulated Wnt-related 'pathways' as reported by GSEA:

Rank	Name	NOM p-val	FDR q-val	FWER p-val
87	REACTOME_SIGNALING_BY_WNT	0.027	0.038	0.772
176	KEGG_WNT_SIGNALING_PATHWAY	0.350	0.583	1.000
194	BIOCARTA_WNT_PATHWAY	0.481	0.672	1.000
199	WNT_SIGNALING	0.518	0.691	1.000
216	ST_WNT_BETA_CATENIN_PATHWAY	0.675	0.801	1.000

B. Top 10 pathways that were 'upregulated' in colon adenoma according to GSEA:

Rank	Name	NOM p-val	FDR q-val	FWER p-val
1	REACTOME_TRANSCRIPTION_OF_THE_HIV_GENOME	0.000	0.024	0.025
2	REACTOME_RNA_POLYMERASE_II_TRANSCRIPTION	0.000	0.018	0.035

3	REACTOME_LATE_PHASE_OF_HIV_LIFE_CYCLE	0.000	0.012	0.035
4	REACTOME_FORMATION_AND_MATURATION_OF_MRNA_TRANSCRIPT	0.000	0.016	0.060
5	KEGG_UBIQUITIN_MEDIATED_PROTEOLYSIS	0.000	0.016	0.071
6	REACTOME_DNA_REPAIR	0.000	0.015	0.079
7	REACTOME_GENE_EXPRESSION	0.000	0.014	0.090
8	KEGG_RNA_DEGRADATION	0.002	0.014	0.096
9	REACTOME_HIV1_TRANSCRIPTION_INITIATION	0.000	0.013	0.099
10	REACTOME_HIV1_TRANSCRIPTION_ELONGATION	0.000	0.012	0.106

C. Top 10 ‘pathways’ that were ‘downregulated’ in colon adenoma according to GSEA:

Rank	Name	NOM p-val	FDR q-val	FWER p-val
1	REACTOME_TCR_SIGNALING	0.016	1.000	0.863
2	BIOCARTA_TFF_PATHWAY	0.025	1.000	0.878
3	REACTOME_INTEGRIN_CELL_SURFACE_INTERACTIONS	0.002	1.000	0.929
4	KEGG_DRUG_METABOLISM_OTHER_ENZYMES	0.008	1.000	0.948
5	KEGG_INTESTINAL_IMMUNE_NETWORK_FOR_IGA_PRODUCTION	0.008	1.000	0.958
6	KEGG_FC_GAMMA_R_MEDIATED_PHAGOCYTOSIS	0.032	1.000	0.959
7	KEGG_GLYCOSPHINGOLIPID_BIOSYNTHESIS_LACTO_AND_NEOLACTO_SERIES	0.010	1.000	0.959
8	REACTOME_GS_ALPHA_MEDIATED_EVENTS_IN_GLUCAGON_SIGNALLING	0.002	1.000	0.969
9	REACTOME_DOWNSTREAM_TCR_SIGNALING	0.045	1.000	0.969
10	BIOCARTA_AKT_PATHWAY	0.027	1.000	0.970

D. PI3K-related pathway analysis with GSEA on GSE8671:

	**Upregulated in colon adenomas**			
Rank	Name	NOM p-val	FDR q-val	FWER p-val
151	REACTOME_PI3K_AKT_SIGNALLING	0.192	0.336	1.000
	**Downregulated in colon adenomas**			

Rank	Name	NOM p-val	FDR q-val	FWER p-val
26	REACTOME_G_BETA_GAMMA_SIGNALLING_THROUGH_PI3KGAMMA	0.018	0.829	0.997
147	REACTOME_PI3K_CASCADE	0.116	0.431	1.000
296	REACTOME_CD28_DEPENDENT_PI3K_AKT_SIGNALING	0.432	0.604	1.000
